# How causal machine learning can leverage marketing strategies: Assessing and improving the performance of a coupon campaign

**DOI:** 10.1371/journal.pone.0278937

**Published:** 2023-01-11

**Authors:** Henrika Langen, Martin Huber

**Affiliations:** 1 University of Helsinki, Faculty of Social Sciences, Economics, Helsinki, Finland; 2 University of Fribourg, Department of Economics, Fribourg, Switzerland; Shandong University of Science and Technology, CHINA

## Abstract

We apply causal machine learning algorithms to assess the causal effect of a marketing intervention, namely a coupon campaign, on the sales of a retailer. Besides assessing the average impacts of different types of coupons, we also investigate the heterogeneity of causal effects across different subgroups of customers, e.g., between clients with relatively high vs. low prior purchases. Finally, we use optimal policy learning to determine (in a data-driven way) which customer groups should be targeted by the coupon campaign in order to maximize the marketing intervention’s effectiveness in terms of sales. We find that only two out of the five coupon categories examined, namely coupons applicable to the product categories of drugstore items and other food, have a statistically significant positive effect on retailer sales. The assessment of group average treatment effects reveals substantial differences in the impact of coupon provision across customer groups, particularly across customer groups as defined by prior purchases at the store, with drugstore coupons being particularly effective among customers with high prior purchases and other food coupons among customers with low prior purchases. Our study provides a use case for the application of causal machine learning in business analytics to evaluate the causal impact of specific firm policies (like marketing campaigns) for decision support.

## 1 Introduction

Over the last two decades, the amount of customer data available to marketers has increased dramatically with new data types such as social media, clickstream, search query and supermarket scanner data on the rise. The increasing availability of customer Big Data has spawned a new stream of literature on machine learning (ML) methods and tools in the field of business and marketing. The ML literature on designing marketing campaigns ranges from research on modelling customer behavior (e.g., [1, 2]), price sensitivity (e.g., [3]) and purchase decisions (e.g., [4]) to studies on the development of personalized product recommendation systems (e.g., [5, 6]), customer churn management (e.g., [7]) and acquisition of new customers (e.g., [8]).

A common feature of these studies is that they are based on predictive ML, i.e., on identifying patterns of variables in the data in order to use them for predicting an outcome of interest (e.g., sales). Predictive ML algorithms use one part of the data to train models that allow predicting the outcome based on patterns found in the data. Then, the other part of the data is used to identify the model with the best performance, where performance is measured by how close the predicted outcomes are to the observed ones. To minimize the prediction error and thereby maximize the predictive power of a model, a predictive ML algorithm trades off bias, i.e., the systematic deviation of the chosen model specification from the true predictive model, and variance, i.e., the sensitivity of the predictions to which data is used for training the model.

Predictive ML models generally do not provide insights about the causal effects of specific variables or interventions (such as a marketing campaign) on the outcome of interest. When multiple variables capture the same relevant predictive feature, i.e., are correlated with that feature, ML algorithms may identify some of these variables as relevant predictors while attaching little importance to others, regardless of the variables’ causal effect on the outcome. For instance, variables that do not directly or only modestly affect the outcome may enter the predictive model as relevant predictors, simply because they are correlated with other variables that actually affect the outcome. For this reason, it may happen that these other variables play little or no role in the predictive model, even though they have a causal impact on the outcome, because they provide little additional information for the prediction. Therefore, predictive ML is generally not suitable for the causal analysis of ‘what if’ questions, such as how a change in a coupon campaign strategy will affect customer behavior, which would be relevant for decision support, e.g., for optimally designing a marketing campaign.

To improve on the shortcomings of predictive ML in evaluating the impact of implementing vs. not implementing a specific intervention, a fast growing literature in econometrics and statistics has been developing so-called causal ML algorithms. In this paper, we demonstrate the application of such methods in the context of business analytics for decision support, namely for evaluating a marketing intervention. More precisely, we make use of the so-called causal forest by Athey et al [9] to assess the causal effect of coupon campaigns, in which customers were provided coupons for different product types, on customers’ purchasing behavior, i.e., the difference in their expected behavior with and without being targeted by a coupon campaign. While predictive ML algorithms are not able to isolate the causal effects of coupons on customers’ purchasing behavior from the influence of background characteristics (e.g., socio-economic characteristics and price sensitivity) which jointly influence coupon reception and purchasing behavior, the causal forest approach can do so under certain assumptions.

One crucial condition is that all variables that jointly affect coupon reception and purchasing behavior are observed in the data and can thus be controlled for. This condition is known as selection-on-observables or unconfoundedness assumption. Under further conditions on the quality of the ML models used in the causal forest for predicting purchasing behavior and coupon reception as a function of the observed variables, the causal forest approach permits evaluating the average impact of the coupons on all customers, as well as across specific subgroups or customer segments (e.g., different age groups). Our results suggest, for instance, a positive overall effect of coupons for drugstore items. For coupons applicable to ready-to-eat food as well as meat and seafood, on the other hand, we do not find a statistically significant overall effect. An analysis of the effect of drugstore coupons across different customer subgroups reveals that these coupons affect customers with high pre-campaign spending as well as low- to middle-income customers particularly strongly.

Furthermore, we apply optimal policy learning based on ML as proposed by Athey and Wager [10], in order to learn from the data which customer segments should be optimally targeted by coupon campaigns such that the overall effect is maximized. In contrast to predictive ML, optimal policy learning (under certain conditions) allows determining the coupon strategy which is most effective in terms of its impact on sales. This is obtained by a data-driven customer segmentation in which only those segments with sufficiently high effects are targeted by a coupon campaign. The estimated optimal policy for coupons applicable to meat and seafood, for instance, suggests that such coupons should be issued to the following three target groups: low-income customers whose pre-campaign spending did not exceed a certain level, middle-to-high-income customers aged 46 years or older who purchased something from the store in the period prior to the campaign, and middle-to-high-income households with at least five members who did not purchase anything from the store in the pre-campaign period.

The paper proceeds as follows. Section 2 outlines the current state of quantitative research in the marketing literature. Section 3 motivates the application of causal ML methods in the field of marketing and discusses the contribution of our study to marketing research. Section 4 introduces and describes the retailer sales data to be analyzed. Section 5 defines the causal effects of interest based on so-called counterfactual reasoning and discusses the conditions required for applying causal ML (such as the selection-on-observables assumption). Section 6 describes the algorithms for causal analysis and optimal policy learning. Section 7 provides the results of the evaluation of the coupon campaigns as well as the optimal coupon allocation. Section 8 concludes.

## 2 Related literature

The impact evaluation of discounts plays a significant role in the earlier marketing literature from the ‘pre-Big-Data era’, see e.g., [11–14] for studies on causal effects of coupon provision. However, the last two decades have seen a surge of predictive ML applications in business analytics, which appear to increasingly dominate causal analysis, also in marketing. In a keyword-search-based literature review, Mariani et al [15] find that the number of publications on predictive ML and Artificial Intelligence (AI) in marketing, consumer research and psychology has grown exponentially in the past decade (2010–21). The systematic literature reviews by Mustak et al [16] as well as Ma and Sun [17] paint a similar picture, with the latter stating that the rise of ML in marketing began with applications of support vector machines, a specific type of ML algorithms. This was then followed by studies introducing text analysis, topic modelling and reinforcement learning into marketing research, as well as by marketing applications of deep learning and network embedding. Questions about the impact of marketing campaigns, the influence of certain external factors on the success of a campaign and the heterogeneity of campaign effects across customer segments appeared to become comparatively less important (see e.g., [17, 18]), even though most recently, the marketing literature saw first applications of causal ML alogithms (such as causal trees).

The following sections summarize the current state of research on discount campaigns using (traditional) causal inference (Section 2.1), predictive ML (Section 2.2) and causal ML 2.3.

### 2.1 Causal inference in marketing

A large number of studies assess the causal effects of marketing campaigns on consumer response. These studies typically rely on (field) experiments or traditional methods for causal inference based on observational data.

Studies on the effectiveness of coupons based on field experiments can be found in the early marketing literature but also more recently. Bawa and Shoemaker [19] assessed the impact of coupons on sales in a field experiment in which coupons for a particular brand were given to both, retail store customers that had previously purchased the advertised brand and to households that had not. Fong et al [20] conducted a field experiment in which they randomly distributed mobile tickets with different discount depths among individuals who were in different designated areas at a given time. They evaluated the effects of discount depth and proximity to the advertised movie theater on the use of these coupons. Other examples of field studies include Taylor and Long Tolbert [21], Heilman et al [22], Venkatesan and Farris [23] as well as Gose et al [24] to name only a few examples.

The field of observational studies on coupons has recently experienced methodological developments towards improved inference methods and a larger awareness of endogeneity issues. Early observational studies on the effects of coupons were frequently based on estimating theoretically derived models of consumer utility considerations, attitudes, and/or behavior using regressions. Sethuraman and Mittelstaedt [25] use aggregate scanner panel data to estimate the impact of coupons for national brands and private labels on the sales share of private label products, leveraging differences in coupon provision across product categories. Srinivasan et al [26] evaluate the effect of exposure to coupons on beverage sales among customers who do not redeem coupons by fitting a regression model to aggregate brand sale and coupon redemption data from three retail stores. They find that exposure to coupons may also increase sales among coupon nonusers. Papatla and Krishnamurti [27] estimate the longer-term effects of coupon promotions on the customers’ loyalty to brands and price sensitivity in the weeks following the coupon campaign. Their study reveals that coupon redemption reduces brand loyalty and increase price sensitivity in the long run. Other regression-based studies on the effect of coupon redemption and/or reception on sales and brand choice include papers by Neslin [28], Raju et al [12], Chiang [29], Dhar and Raju [30] and Sun [31].

Rubin and Waterman [32] apply propensity score matching to evaluate the effect of marketing interventions aimed at physicians in order to promote the prescription of a ‘lifestyle’ drug. They also rank the physicians according to their estimated expected individual-level effects, which in turn can be used to derive a tailored marketing strategy. Li et al [33] propose an estimator that combines dimensionality reduction and nearest-neighbor matching to estimate the effect of high discount promotions in promotional emails on customers’ response to those emails. Among others, Berning and Zheng [34], Venkatesan and Farris [23] as well as Danaher et al [35] apply instrumental variable approaches to investigate exposure and/or redemption effects of coupon campaigns by isolating exogenous variation in coupon provision or redemption.

Dafny et al [36] assess the effect of co-payment coupons, i.e., coupons issued by pharmaceutical companies that reduce insured patients’ out-of-pocket costs for promoted drugs, on the patients’ decision between brand-name drugs and bioequivalent generics by means of difference-in-differences as well as triple-difference models. They find that co-payment coupons significantly increase sales of branded products while reducing the sales of bioequivalent generics. Reimers and Xie [37] assess the effect of e-coupon provision on alcohol sales by means of a difference-in-differences approach, exploiting the fact that the restaurants in their sample issued e-coupons at different points in time. They find that the provision of e-coupons increases demand for alcoholic beverages both during and after the promotion period. Guan et al [38] use a difference-in-differences approach to evaluate whether providing coupons to retail stores leads to changes in food purchases from before to after the campaign, and find that providing coupons significantly increases food purchases, especially purchases of ready-to-eat foods. Other studies analyze the effect of a farmers’ market coupon initiative on fruit and vegetable purchases in low-income neighbourhoods [39], that of coupons and other social media marketing tools on product sales [40], and that of coupons distributed by a hair salon on customer spending per visit, frequency of visits, and attrition [41].

Another domain of the marketing literature investigates the heterogeneity of marketing effects across customer characteristics and the circumstances under which customers are targeted by coupon and other promotional campaigns. Among them, Gopalakrishnan and Park [42] analyse whether high- and low-consumption customers, as defined by their purchasing behavior during the 12 months prior to the experiment, differ in their responsiveness to coupon campaigns. Andrews et al [43] study whether the level of occupancy (or crowdedness) of a subway affects passengers’ response to mobile advertising campaigns and find a statistically significant positive association. Based on a field experiment, Spiekerman et al [44] conclude that proximity to the location for which coupons are distributed influences coupon redemption, and that this association is much more pronounced in the city center than in suburban areas.

Furthermore, several studies evaluate how certain configurations of coupons, such as face value, distribution method and expiry date, affect consumer behavior. The experimental studies by Zheng et al [45] as well as Biswase et al [46] assess how the size of discounts affects consumers’ perceptions of product quality and purchase intentions. Leone and Srinivasan [13] use supermarket scanner data to analyze the effect of coupon face value on sales and profits, while Anderson and Simester [47] study the long-term effects of discount size on the purchasing behavior of new and established customers in an experimental setting. Other contributions as e.g., [11, 14, 42, 48, 49] analyze how further aspects of coupon and discount campaign design affect consumer behavior.

### 2.2 Predictive ML in marketing

In recent years, many studies have focused on ML-based prediction of coupon redemption and associated sales. They use ML algorithms to model customer behavior as a function of customers’ previous transactions, their response to past coupon/discount campaigns and their socio-economic characteristics in order to predict the likelihood of customers to redeem coupons or take up discounts and make purchases.

Pusztová and Babič [50] as well as He and Jiang [51] compare the performance of different ML-based classification algorithms in predicting coupon redemption in digital marketing campaigns. The former study concludes that so-called Support Vector Machines provide the most accurate predictions, while the latter study finds that the gradient boosting framework ‘XGBoost’ performs best. Greenstein et al [52] introduce an algorithm that combines co-clustering and random forest classification to predict redemption of mobile restaurant coupons based on demographic and contextual variables such as the consumer’s distance to the restaurant relative to the size of the coupon discount. Ren et al [53] develop a two-stage model for estimating the probability of coupon redemption. In the first stage, customers are clustered based on their past purchase and redemption behavior, while the second stage consists of fitting prediction models for the different customer clusters. Furthermore, several studies such as [45, 54, 55] predict customer behavior in the context of coupon or other discount campaigns by means of several ML methods.

### 2.3 Causal ML in marketing

The rise of predictive ML has prompted e.g., Anderson [56], Lycett [57] and Erevellese et al [58] to argue that theory-based causal inference has lost some of its relevance for business decisions in the light of the large data sets and sophisticated predictive ML methods available to marketers today. However, these views were soon challenged in several studies that emphasize the importance of causal reasoning and risks of basing decisions solely on correlations, see e.g., [59, 60]. More recently, a growing number of contributions have stressed the importance of integrating ML and causal inference, see e.g., [18]. Among them is Hunermund et al [61], who investigate the use of causal methods in business analytics by combining qualitative interviews and quantitative surveys among data scientists and managers in a mixed-methods research design. They document an ongoing shift in corporate decision making away from an exclusive focus on predictive ML and towards the use of causal methods, based on both observational and experimental data.

Recently, the use of causal ML in marketing research appears to be increasing. To the best of our knowledge, however, there are virtually no studies that evaluate the causal effect of coupon campaigns on customer behavior using causal ML, as we do in this paper. Smith et al [62] use predictive ML for deriving optimal coupon targeting strategies and estimate the profits that would accrue under those strategies out of sample, i.e., in parts of the data not used for deriving the strategies. The estimations make reference to the so-called potential outcomes framework for defining causal effects when applying causal inference or causal ML. However, the study by Smith et al [62] is conceptually different from ours in that it uses the potential outcomes framework to compare coupon targeting strategies inferred from different predictive ML algorithms, while we apply a causal ML algorithm, namely the optimal policy learning approach of Athey and Wager [10], to derive the optimal coupon targeting strategy.

One study in the field of marketing which does consider causal ML is [63]. They assess the performance of so-called Double ML, see [64], and propensity score matching, see [65], for estimating the causal effect of conversion ads on Facebook. Such ads aim to increase online activity like page visits, sales and views on an external website. For their analysis, the authors take advantage of the fact that Facebook offers businesses the opportunity to assess their ad campaigns by means of randomized experiments. Gordon et al [63] compare the effect estimates based on Double ML and propensity score matching with those from the experiments, finding that Double ML outperforms propensity score matching, but that both approaches overestimate the effect substantially. This highlights the importance of observing and appropriately controlling for all factors jointly affecting the intervention and customer behavior when causally assessing marketing interventions. Also Huber et al [66] consider Double ML when analyzing observational data to investigate whether discounted tickets induce Swiss railway customers to reschedule their journeys, e.g., to shift demand away from peak hours.

Narang et al [67] apply causal forests, the causal ML framework developed by Wager and Athey [68] as well as Athey et al [9] also used in this study (see Section 6), to assess the heterogeneity across shoppers in how mobile app failures affect the frequency, volume, and monetary value of their purchases. Guo et al [69] assess the effect of a law requiring pharmaceutical firms to disclose their marketing payments to physicians on the firms’ payments to physicians using a Difference-in-Differences approach and assess expected individual-level effect heterogeneity by means of causal forests. Zhang and Luo [70] incorporate causal forests in their study on modelling restaurant survival as a function of photos posted on social networks. They find that the total volume of user-generated content and the extent to which user photos are rated as helpful have a significant positive effect on the likelihood of restaurant survival. Another study from the domain of marketing that uses causal ML is Cagala et al [71]. The authors estimate the optimal strategy for distributing gifts among potential donors in a fundraising campaign that maximizes expected net donations. They find that the identified optimal targeting rule outperforms different non-targeted gift distribution rules, even when the optimal targeting rule is estimated based only on publicly available geographic information or on data from a previous fundraising campaign conducted in a similar sample.

In addition to the causal ML applications outlined here, there is another strand of literature that aims to explain AI-generated decisions, namely eXplainable Artificial Intelligence (XAI). The field of XAI encompasses artificial intelligence (AI) methods that generate explanations for decisions made by AI in order to make them understandable to humans. The standard approach is to generate business decisions for the problem at hand using AI and to create a second, but interpretable model that approximates the original “black-box” model which produced the decision (see, for example, [72]). XAI applications in the field of marketing include studies by Haag et al [73] and Marín Díaz [74].

## 3 Context and contribution

In the following, we will use coupon promotions as a running example to highlight the merits of causal ML in business analytics and marketing research. In the context of coupon campaigning strategies, marketers are arguably interested not only in predicting customer behavior, but also in measuring the causal effects of alternative campaigns on customer behavior. Such effects correspond to the difference in the customers’ (average) behavior when being vs. not being addressed by a particular campaign. Intuitively, this requires comparing a customer’s observed behavior under the actual assigned coupon with the potential (and not directly observed) behavior that would have occurred had coupon provision been different from that actually observed, an approach commonly referred to as counterfactual reasoning. Such a causal assessment is necessary for determining whether and to which extent a campaign is effective in altering customer behavior and for understanding how customer behavior would change if coupons were distributed differently.

Predictive ML models, however, do not inform about causal effects of campaigns. In a predictive ML model, the predictive power of coupon provision on customer behavior generally does not correspond to such a causal effect, because it is affected by so-called regularization bias. The latter arises because ML algorithms generally shrink the importance of (some or all) predictors in order to reduce the variance of prediction, ideally to improve predictive performance. Regularization bias may occur, for instance, when coupon provision is strongly correlated with other (good) predictors (such as previous purchases) and/or when its effect on consumer behavior is comparably small, so that coupon provision has little predictive value. A further issue is selection bias, meaning that coupons may pick up the effects of other variables whose importance has been shrunk by the ML algorithm. The implementation of coupon campaigns should be based on estimations of causal effects that avoid regularization and selection bias, as is the case with causal ML algorithms such as DML and causal forests, if certain conditions hold.

The importance of estimating the causal effect of coupon campaigns in the context of decision support, rather than merely predicting customer behavior, can be illustrated by means of a simple example. Suppose a retailer estimates a model to predict sales based on observational data from a previous coupon campaign in which (in an attempt to re-activate dormant customers) coupons were distributed primarily among customers who had not been in the store for a while, rather than among frequent shoppers. The estimated predictive model might indicate a negative association between coupon provision and sales, since the coupon campaign is likely to re-activate only some inactive customers, so that the (formerly) inactive customers on average purchase less than the frequent shoppers. The true effect of receiving a coupon, however, might actually be positive. A positive effect implies that when comparing two groups of (formerly) inactive customers with comparable background characteristics (like willingness to buy), where the first receives coupons while the second does not, the average purchases of the first group are higher. The predictive model therefore confuses (or confounds) the causal effect of the coupon campaign with that of being a dormant vs. a frequent shopper, thus incorrectly pointing to a negative effect.

In a second scenario, the retailer decides to issue coupons in the store. This way, frequent shoppers are regularly provided with coupons, while dormant customers rarely if ever receive any. A predictive model now detects a positive relationship between the provision of coupons and sales, although the actual effect of providing coupons could be negative, namely if frequent customers use the coupons for products they would have bought anyway. If the campaigns were evaluated using predictive methods and the results were misinterpreted as causal, marketers would come to the conclusion that the first campaign was ineffective while the second was effective. Causal methods, on the other hand, enable marketers under certain conditions to control for such biases (e.g., due to differences in purchasing behavior between frequent and dormant customers) and to consistently estimate the effect of coupon campaigns. Further, these methods can also be applied to assess effect heterogeneity and identify an optimal coupon distribution scheme (or policy) that targets those customers whose average purchases are sufficiently responsive to receiving a coupon.

In causal studies on discounts, the impact of providing coupons is typically assessed either based on random experiments or observational data from previous campaigns when controlling for observed characteristics (or covariates) that are likely to be associated with both coupon provision and consumer behavior. Traditional, non-ML-based causal inference methods require the researcher or analyst to manually select covariates based on theoretical considerations, domain knowledge, intuition and/or previous empirical findings. Examples for such covariates in the context of campaign evaluations include past purchasing behavior, exposure to previous campaigns, and socio-economic characteristics such as age, gender, or income. Manual selection of covariates entails the risk of omitting important control variables and may even be practically infeasible in Big Data contexts with a very large set of potential covariates (e.g., collected from online platforms), including unstructured data containing, e.g., text or clickstreams. Furthermore, conventional causal inference methods require the researcher to specify how, i.e., through which functional form (like, e.g., a linear model), the selected covariates are associated with coupon provision and purchasing behavior.

Causal ML methods, in contrast, permit taking advantage of the full amount of information in the data to detect relevant covariates (which have an important influence on coupon provision and consumer behavior) in a data-driven way and to control for them, as well as to flexibly estimate the functional form of statistical associations. Still, the observational data have to meet certain conditions, as described in Section 5.2.

We assess the causal effect of receiving coupons on customer spending by means of the causal forest, a causal ML method developed by Wager and Athey [68] that draws on the ML technique of random forests. Just as the random forest algorithm, the causal forest approach draws random subsamples from the data and then creates decision trees in each subsample using a random set of observable variables. To grow these trees, the observations are divided into two subgroups at different values of the variables under consideration, and the algorithm selects the split that fulfils a given splitting criterion. The same is repeated in each of the resulting subsamples, which are commonly referred to as nodes, until a pre-defined stopping rule is met. Finally, the algorithm averages over all trees.

Causal and random forests differ with regard to the splitting criteria they employ to split a sample based on the observed variables, while both methods rely on averaging the respective splitting approach over many samples, which are repeatedly drawn from the original data. The random forest algorithm aims to learn a model that can predict an outcome based on covariates. It therefore splits the covariate space such that the residuals between the observed outcomes and the mean outcomes within subgroups resulting from that split is minimized. Thus, the mean outcome in a subgroup serves as prediction for the outcome of all observations in that subgroup (or node). The causal forest approach, on the other hand, aims at determining splitting rules that maximize the heterogeneity of estimated treatment effects between nodes. Therefore, the algorithm focusses on the conditional average treatment effect (CATE) rather than on the conditional average outcome as splitting criterion and chooses the splitting rule such that the estimated effects differ most between the resulting nodes. The CATE estimates ultimately provided by the causal forest (by averaging over many samples) are also known as individualized effect estimates, as they are a function of an individual’s covariates. This permits assessing the effect heterogeneity of coupon provision across customer characteristics and campaign periods.

Like other causal ML methods, the causal forest approach combines effect estimation based on so-called Neyman [75]-orthogonal scores with sample splitting for the estimation of the CATEs in nodes defined upon the covariates. Put simply, methods based on orthogonal scores first predict treatment and outcome models as functions of the covariates and then use these models to ‘purge’ the influence of the covariates from the treatment and the outcome before estimating treatment effects. In this process, the treatment and outcome models, which are often referred to as plug-in parameters for the effect estimation to follow, can be learnt by ML algorithms. The purpose of purging any influence of the covariates from both the treatment and the outcome (a process called orthogonalization) is to make effect estimation more robust to approximation errors in the prediction of either plug-in parameter. Such approximation errors may arise from ML-related regularization biases, i.e., from neglecting less predictive covariates of the treatment and/or outcome in the estimation of the respective plug-in parameters.

Sample splitting, on the other hand, aims to avoid overfitting in the estimation of CATEs, i.e., fitting the effect heterogeneity model too strongly to the particularities of the estimation sample, such that the causal forest picks up not only the actual differences of causal effects across covariates, but also random noise. In order to prevent such overfitting bias, the random sample used for learning a causal tree is itself randomly split into two subsamples, one for building the tree by following the procedure mentioned above, while the other one is used for estimating the treatment effect in every node of the learnt causal tree. That is, by following the splitting rules learnt in the first subsample, the algorithm populates the nodes of the estimated tree with the observations from the second subsample and calculates the CATE in each node based on the observations that fall into the respective node. Trees that are estimated based on this sample splitting procedure are commonly referred to as ‘honest’ trees (because they avoid overfitting) (see Section 6.1 for a more technical discussion of the causal forest algorithm as implemented in our application).

While the causal forest framework is well-suited for investigating treatment effect heterogeneity, it also permits estimating the average treatment effect (ATE) of coupon provision, which again relies on combining effect estimation based on Neyman [75]-orthogonal scores with sample splitting. This ensures that the ATE is estimated with $\sqrt{n}$-consistency, i.e., the ATE estimate converges to the true ATE with a convergence rate of $1/\sqrt{n}$, provided that the ML steps satisfy specific regularity conditions (like *n*^−1/4^-consistency). Section 6.2 describes the procedure for ATE estimation of coupon reception used in our application.

The argument for counterfactual reasoning further above also applies to the objective of optimizing the distribution of coupons across customer segments, i.e., optimal policy learning, as discussed in [76–79]. Basing optimization on predictive ML models, as advocated in several studies on predicting coupon redemption (e.g., [52–54]), ignores the fact that predictive models do generally not provide information on causal effects and their heterogeneity across different customer segments. Optimal policy learning as suggested by Athey and Wager [10], on the other hand, is a causal ML approach for determining treatment rules which ensures that those customers for whom the largest effects can be expected are targeted by the campaign. In Section 6.4, we demonstrate the use optimal policy learning for detecting customer segments that should or should not be targeted by coupon campaigns in order to maximize the effectiveness of these campaigns.

## 4 Data

In our empirical application, we analyze sales data on coupon campaigns of a retailer provided as part of the AmExpert 2019—Machine Learning Hackathon [80]. The data set contains information on socio-economic characteristics of retail store customers, coupons received during the campaigns as well as coupon redemption and purchasing behavior. The retail store ran several campaigns issuing coupons with discounts for certain products, with some coupons being applicable to individual products only and others to a range of products. In each of the 18 partially time-overlapping campaigns falling into the time span covered by the data set, the store distributed 1 to 208 different coupon types each applicable to up to 12,000 products, most of which belong to the same product category. The coupons were distributed in such a way that each customer received 0 to 37 different coupons per campaign with the composition of this set of coupons varying between the recipients. Apart from the information on provided coupons, the data set contains details on all purchases made by each registered customer between January 2012 and July 2013, including the date of the transaction, the redeemed coupons, the product type of each purchased product and the price paid.

For our analysis, we group the coupons into five broad categories mirroring the products they can be used for. More concisely, we distinguish between coupons applicable for ready-to-eat food items, meat and seafood, other food, drugstore items and other non-food products. One could arguably also be interested in more fine-grained coupon categories or in a paricular coupon or discount type rather than in our broader coupon categories, which would, however, require a larger data set to obtain satisfactory statistical power. Due to the temporal overlap of campaign periods, we need to redefine them such that each of the resulting artificially generated campaign periods coincides with the validity period of a given set of coupons. That is, all coupons which are valid in some artificial campaign period are valid during the entire period. By doing so, we can fully attribute changes in purchasing behavior from one artificial campaign period to another to the coupons valid in the respective periods. From now on, the 33 newly defined artificial campaign periods will simply be referred to as campaign periods. To account for differences in the duration of campaign periods, we consider the average per-day expenditures per customer and campaign period as our outcome of interest. For estimating the causal effect of coupon provision on the buying behavior, we pool the customer-specific purchases across campaign periods, yielding 33 observations per customer.

Table 1 provides some descriptive statistics for our data, namely on observed customer characteristics, the share of coupons redeemed and daily in-store spending (descriptive statistics on the composition of daily expenditures by product type are provided in S1 Table). The table reports the mean of these variables in the total sample of 50,624 observations, as well as among observations that received a coupon and among those that did not. Further, it contains the mean difference in these variables between coupon receivers and non-receivers, as well as the p-value of a two-sample t-test. In some 30% of the observations, customers received at least one coupon. Furthermore, customers who received a coupon had on average higher expenditures in the retail store than customers who did not, suggesting that the retailer did not target its previous campaigns to re-activate dormant customers.

**Table 1:** Descriptive statistics.

*variable*	Overall	Coupon Receivers	Non-Receivers	Diff	p-val
	N = 50,624	N = 15,327	N = 35,297		
*daily expenditures*	202	245	184	61	0
*age: 18–25*	0.028	0.031	0.027	0	0.02
*26–35*	0.082	0.102	0.074	0.03	0
*36–45*	0.118	0.141	0.108	0.03	0
*46–55*	0.171	0.191	0.163	0.03	0
*56–70*	0.037	0.045	0.034	0.01	0
*70+*	0.043	0.039	0.045	-0.01	0
*unknown*	0.52	0.451	0.55	-0.1	0
*family size: 1*	0.157	0.171	0.15	0.02	0
*2*	0.192	0.213	0.182	0.03	0
*3*	0.066	0.079	0.06	0.02	0
*4*	0.03	0.04	0.026	0.01	0
*5+*	0.036	0.047	0.031	0.02	0
*unknown*	0.52	0.451	0.55	-0.1	0
*marital status: married*	0.2	0.234	0.186	0.05	0
*unmarried*	0.072	0.084	0.067	0.02	0
*unknown*	0.728	0.682	0.747	-0.07	0
*dwelling type: rented*	0.026	0.033	0.023	0.01	0
*owned*	0.454	0.516	0.428	0.09	0
*unknown*	0.52	0.451	0.55	-0.1	0
*income group: 1*	0.037	0.042	0.035	0.01	0
*2*	0.043	0.051	0.04	0.01	0
*3*	0.044	0.049	0.042	0.01	0
*4*	0.104	0.113	0.1	0.01	0
*5*	0.118	0.137	0.11	0.03	0
*6*	0.056	0.061	0.053	0.01	0
*7*	0.02	0.023	0.019	0	0.01
*8*	0.023	0.03	0.021	0.01	0
*9*	0.018	0.024	0.016	0.01	0
*10*	0.006	0.006	0.006	0	0.89
*11*	0.003	0.003	0.003	0	0.43
*12*	0.006	0.01	0.005	0	0
*unknown*	0.52	0.451	0.55	-0.1	0
*coupons redeemed*		0.030			

Mean of the variables in the total sample (’Overall‘), among coupon receivers and non-receivers as well as the mean difference across treatment states and the p-value of a two-sample t-test.

We also see that the retailer does not have information on the socio-economic characteristics of all customers in the registry, but only for about half of them, as the corresponding variable values are unknown for many observations (see the coding ‘unknown’). Such a high rate of non-response in variables can entail selection bias when estimating the effects of interest. For this reason, we will conduct several robustness checks in the empirical analysis to follow further below (see Section 7.5). The descriptive statistics also reveal that some socio-economic characteristics as well as their observability are correlated with the reception of coupons. For example, customers aged 70 years or older are less likely to be targeted by a coupon campaign. The main difference in the likelihood of receiving a coupon seems to be between customers whose socioeconomic characteristics are not available and those whose characteristics are known, with the former less likely to receive a coupon.

As is noted in several studies (e.g., [35, 44]) coupon redemption rates are typically low, not exceeding 1 to 3% on average. This is also the case in our data, as only in 3% of the observations of coupon recipients did they actually redeem a coupon. However, as mentioned further above, coupons may not only influence customer behavior when redeemed, but may also serve as an advertising tool which attracts customers to the store even without them redeeming the coupon.

## 5 Identification

### 5.1 Definition of causal effects

We are interested in estimating the causal effect of a specific intervention, commonly referred to as ‘treatment’ in causal analysis and henceforth denoted by *D*, on an outcome of interest, denoted by *Y* (throughout this paper, capital letters denote random variables and small letters specific values of random variables). In our context, *D* reflects the reception or non-reception of coupons and *Y* the purchasing behavior, measured as the average per-day expenditures during the coupon validity period. In the simplest treatment definition, *D* is binary and takes the value 1 when the respective customer is provided with a coupon and 0 if this is not the case. Mathematically speaking, the value *d* which treatment *D* can take satisfies *d* ∈ {0, 1}. The set of observations with *d* = 1 is commonly referred to as the treatment group, those for which *d* = 0 are called control group. Our subsequent discussion of causal effects and the statistical assumptions required for their measurement will focus on this binary treatment case for the sake of simplicity. However, our empirical analysis will also separately consider the effects of receiving coupons for five product categories, by running separate estimations for the comparison of each category to not receiving any coupons. This implies that the assumptions introduced in Section 5.2 need to hold for each of these categories. For discussions of multi-valued treatments, see e.g., [81, 82].

For defining the causal effect of coupon provision, we rely on the potential outcome framework pioneered by Neyman [83] and Rubin [84]. Let *Y*(*d*) denote the potential (rather than observed) outcome under a specific treatment value *d* ∈ {0, 1}. That is, *Y*(1) corresponds to a customer’s potential purchasing behavior if she received a coupon, while *Y*(0) is the behavior without a coupon. The causal effect of the coupon thus corresponds to the difference in the purchasing behavior with and without coupon, *Y*(1) − *Y*(0), but can unfortunately not be directly assessed for any customer. This is due to the impossibility of observing customers at the same point in time under two mutually exclusive coupon assignments (1 vs. 0), which is known as the ‘fundamental problem of causal inference’, see [85]. This follows from the fact that the outcome *Y* which is observed in the data corresponds to the potential purchasing behavior under the coupon assignment actually received, namely *Y* = *Y*(1) for those receiving a coupon (*d* = 1), and *Y*(0) = *Y* for those who do not (*d* = 0). For coupon recipients, however, *Y*(0) cannot be observed in the data, while for customers without a coupon *Y*(1) remains unknown.

Even though causal effects are fundamentally unidentifiable at the individual level, we may, under the assumptions outlined further below, evaluate them at more aggregate levels, i.e., based on groups of treated and nontreated individuals. One causal parameter which is typically of crucial interest is the average causal effect, also known as average treatment effect (ATE), i.e., the average effect of coupon assignment *D* on purchasing behavior *Y* among the total of customers. Formally, the ATE, which we henceforth denote by Δ, corresponds to the difference in the average potential outcomes *Y*(1) and *Y*(0):

$$\begin{array}{c} \Delta =E[Y(1)-Y(0)], \end{array}$$ (1)

where ‘*E*[…]′ stands for ‘expectation’, which is the average in the population.

### 5.2 Discussion of identifying assumptions

In order to identify the ATE defined in the previous section, we need to impose several identifying assumptions, which are outlined in this section. We note that in the subsequent discussion, ‘⊥’ stands for statistical independence. Further, *X* denotes the set of covariates that should not be affected by treatment *D* and therefore be observed before or at, but not after, treatment.

**Assumption 1 (conditional independence of the treatment)**:

*Y*(*d*)⊥*D*|*X* for all *d* ∈ {0, 1}.

Assumption 1 states that the treatment is conditionally independent of the outcome when controlling for the covariates, and is known as ‘selection on observables’, ‘unconfoundedness’ or ‘ignorable treatment assignment’, see e.g., [65]. The assumption implies that there are no unobservables jointly affecting the treatment assignment and the outcomes conditional on the covariates. This condition is satisfied if the coupons are quasi-randomly distributed among observations with the same values in *X*. The retailer may therefore base the distributing of coupons on customer or market characteristics observed in the data, however, not on unobserved characteristics that affect purchasing behavior even after controlling for the observed ones.

We control for the variables in Table 1, period fixed effects, the customers’ average daily pre-campaign spending by product category, as well as for the coupons she received and redeemed in the period prior to the campaign. When evaluating the effect of specific coupon categories, we also include dummies that indicate whether a customer received coupons from another category at the moment of treatment assignment. This is because the availability of other coupons influences purchase behavior and is likely to be correlated with the probability of receiving coupons of the category under study. The reason for including period fixed effects is that there is no information available on holidays or weekdays on which the store is closed or has shortened opening hours, that is, circumstances that may affect purchasing behavior. Also, the retailer is likely to distribute coupons differently across campaign periods. Including pre-campaign expenditures allows controlling for general differences in purchasing behavior between customers that might be correlated with the likelihood of receiving coupons, since the retailer presumably bases decisions about whom to allocate which coupon(s) on past purchasing behavior.

The covariates considered in our estimation are similar to those included in studies on the effect of coupon campaigns that rely on traditional causal inference approaches, see, e.g., [86, 87]. In both studies, the covariates contain demographic characteristics as well as a proxy for the customers’ economic situation and their purchasing behavior prior to the coupon campaign. Unlike the methods used in these studies, however, the causal ML approach applied here permits controlling for covariates in a flexible, possibly non-linear way, and does not require pre-selecting variables. Studies on predicting coupon redemption by ML frequently rely exclusively on customer behavior and coupon characteristics as predictors of coupon redemption, while not including socio-demographic characteristics of customers, see e.g., [52] use and [51].

In their study on the performance of causal ML in evaluating Facebook ads, Gordon et al [63] include users’ gender, age and household size but—unlike our data—their data lack information on users’ economic situation, such as their income, employment status, or wealth. They also use several Facebook-specific covariates measuring users’ activity on Facebook (likes, posts, type of device used and interests explicitely expressed on Facebook). Furthermore, they take into account users’ response to earlier ads from other companies, which is comparable to the covariates on pre-campaign purchasing behavior, coupon reception and coupon redemption considered in our analysis.

Despite the large differences in the amount of information available in the Facebook study and our analysis, we cannot automatically conclude that the set of covariates in our analysis is insufficient for satisfying Assumption 1. In fact, the algorithms used by Facebook to determine the target audience for ad placement might be more complex and information-hungry than a retailer’s coupon strategy. In order to properly assess the causal effect of Facebook ads, any of this information incorporated in Facebook’s ad placement algorithms that also drives the outcome of interest, namely customer response, would need to be controlled for. Likewise, our evaluation needs to control for outcome-relevant information based on which the retailer determined the coupon assignment. If the retailer based the distribution of coupons systematically on covariates observed in its customer database (but not on other factors), then Assumption 1 holds in our context.

**Assumption 2 (common support)**:

0 < *Pr*(*D* = 1|*X*)<1.

Assumption 2 states that the conditional probability of being treated given *X*, in the following referred to as the treatment propensity score, is larger than zero and smaller than one. This so-called common support condition implies that for all values the covariates might take, customers have a non-zero chance of being treated and a non-zero chance of not being treated. While this assumption is imposed w.r.t. to the total of a (large) population, meaning that both treated and non-treated customers exist conditional on *X*, we can and should also verify it in the data. In our sample, common support appears to be satisfied, as there exist no combinations of covariate values for which either only customers with coupons (of a certain category) or no coupons exist. S1–S3 Figs show the distribution of the estimated propensity scores for receiving coupons (of a particular type) among recipients and non-recipients of that particular coupon(s). The distributions overlap (although the overlap is partially thin), i.e., for each observation in one group, observations can be found in the other group that are comparable with respect to the propensity score.

Another condition that needs to be satisfied is the so-called Stable Unit Treatment Value Assumption (SUTVA), see e.g., [88]. In our context, SUTVA rules out that the coupons provided to one individual affect the potential outcome of another individual. The assumption that there are no inter-personal spillover effects of coupon campaigns may be problematic in our setting. Customers receiving coupons may induce their peers to make purchases by, for instance, telling peers about the products they bought when redeeming the coupon or by visiting the store together with peers. On the other hand, customers with coupons may also redeem their coupons to buy the coupon-discounted products not only for themselves but also for their peers, thereby reducing the purchases made by their peers. Such scenarios appear particularly likely when there are several members of the same household in the customer base. There is ongoing research on how to deal with such SUTVA violations under certain assumptions like the observability of groups affected by spillovers, see e.g., [89–95]. However, in our data set, the relationships between customers are not observable, meaning the data does not allow accounting for possible spillovers of providing coupons to one customer on the outcomes of other customers. If such spillovers existed in our case, they could entail an under- or overestimation of the effect of coupons on purchasing behavior, depending on whether the spillovers occur primarily through treated customers inducing non-treated peers to make purchases or through treated customers redeeming coupons to purchase products for their peers, with the former entailing an overestimation of the outcome under non-treatment and the latter leading to an underestimation.

SUTVA also requires that for every individual in the population, there is a single potential outcome value associated with each treatment state, meaning that there are no different versions of the coupons leading to different potential outcomes. In many empirical applications, it appears likely that at least some aspects of SUTVA are violated, and for this reason, there exist several relaxations of this assumption. In our case, the requirement that there be no different treatment versions is particularly problematic given that we group different coupons into broader categories. The treatment of being provided with coupon(s) from one category comprises the receipt of different coupons, each applicable to a distinct set of products from the respective product category. If a customer is not equally interested in all products belonging to that product category, the customer may only redeem a coupon and/or change her purchasing behavior if the coupon is applicable to certain products. For this reason, we are in a setting where there are different treatment versions, each possibly associated with a different potential outcome.

VanderWeele and Hernan [96] relax the original SUTVA by allowing for the existence of different unobservable versions of the treatment as long as there are no different versions of non-treatment and the treatment versions are assigned randomly conditional on the covariates *X*. This permits assessing the average effects of certain bundles of coupons (rather than specific coupons as under the original SUTVA) vs. not receiving any coupons. Indeed, the assumption that there is only one version of non-treatment is satisfied in our analysis of the effect of receiving some vs. no coupons, under the assumption that the marketer has not run any undocumented discount campaigns during the study period. Furthermore, when assessing the effects of coupons applicable to specific product categories, we control for all other coupons that each customer received at treatment assignment, which in turn creates non-treatment states that are necessarily equal after controlling for other coupons. Table 1 and S2 Table show that the coupons were distributed under consideration of the covariates in the customer registry. We must now assume that the propensity of receiving a coupon (version) differs only depending on observed characteristics, but not on characteristics that are not available to us. This issue can be easily circumvent in practice as long as the information on customers available to marketing campaign planners is also available to those evaluating the campaign.

### 5.3 Inter-temporal spillover effects

While our main analysis focusses on the (short-term) effect of coupon provision on purchasing behavior during the validity period of the coupon (rather than longer-term coupon-induced behavioral shifts), our framework does not rule out the existence of inter-temporal spillover effects on customers’ purchasing behavior. By including pre-campaign coupon reception and redemption as control variables, we aim at controlling for the fact that previous coupons may influence customer behavior in the current outcome period. As a further inter-temporal effect, however, customers may advance their purchases planned for later periods towards a more recent campaign period in which they receive coupons applicable to the products they are interested in. This needs to be kept in mind when interpreting the effects.

In order to get an impression of the extent to which coupons induce inter-temporal spillover effects and, on the other hand, longer-term increases in customer retention, we also assess the effect of coupon provision in campaign period *t* on daily expenditures in subsequent periods, namely in *t* + 1 and *t* + 2. It should, however, be noted that the estimated effect is the total effect of coupon reception on purchasing behavior in these subsequent periods. That is, it does not only capture the longer-term coupon-induced change in purchases at the store (net of spillovers from advancing purchases in periods in which the customer has applicable coupons). Rather, it also captures how coupon provision in *t* affects purchasing behavior in *t* + 1 and *t* + 2 through changing the likelihood of coupon reception in these later periods (e.g., because the customer redeems coupons in *t* or the coupons incentivize her to increase her purchases in *t*). Disentangling the direct effect of coupon provision on purchasing behavior in subsequent periods from the indirect effect mediated via increasing the likelihood of coupon provision in these later periods would require estimating dynamic treatment effects of treatment sequences, such as the sequence of coupon reception in *t* and non-reception in *t* + 1 (see [97] for an approach to estimating dynamic treatment effects by means of DML).

Furthermore, it is worth noting that some coupons valid in *t* may still be valid in *t* + 1 and even *t* + 2. The estimated effect of coupon provision in *t* on purchasing behavior in later periods therefore also partially captures the treatment effect of coupons during their validity period. A look at the data shows that the likelihood of having a valid coupon in *t* + 1 or *t* + 2 is highly correlated with that of having a coupon in *t* (conditional on *X*), be it due to the effect of coupons on re-provision or because the validity period of coupons exceeds that of the artificially created campaign periods. Part of the estimated longer-term effect is therefore likely attributable to the indirect effect of coupon provision in *t* on daily expenditures in *t* + 1 and *t* + 2, via increasing the probability that the customer has valid coupons in these subsequent periods.

## 6 Empirical strategy

In the following, let *i* ∈ {1, …., *n*} be an index for the *n* = 1, 582 customers in the data set and *t* ∈ {1, …, *T*} with *T* = 32 an index for the campaign period. Then, {*Y*_*i*,*t*_, *D*_*i*,*t*_, *X*_*i*,*t*_} denote the outcome, the treatment and the covariates, respectively, for individual *i* in campaign period *t*. Treatment *D*_*i*,*t*_ is a binary indicator measuring exposure to a coupon campaign (of a specific type) and *Y*_*i*,*t*_ denotes the outcome, defined as average per-day expenditures of customer *i* in period *t*. The covariates *X*_*i*,*t*_, all measured prior to or at the time of treatment assignment, include socio-economic variables (see Table 1), the average daily spending by product type in the period prior to the campaign *t* − 1, and variables that measure both whether the customer received coupons in *t* − 1 and whether he/she redeemed any. For estimating the effect of a particular coupon type, *X*_*i*,*t*_ also contains variables on what other coupon types were provided to the customer in *t*; in addition, it includes information not only about whether the customer received coupons in *t* − 1, but also about what type of coupons.

Under the identifying assumptions outlined in Section 5.2, the ATE Δ defined in Eq (1) corresponds to *θ*:

$$\begin{array}{c} \theta =E[E[Y|D=1,X]-E[Y|D=0,X]] \end{array}$$ (2)

As the functional form of *E*[*Y*|*D*, *X*] is unknown, we use the causal forest approach by Wager and Athey [68] to estimate *θ* in a data-driven way rather than regressing *Y* on *D* and *X* based on a parametric model. The causal forest algorithm learns models for predicting the average of *Y* as a function of *X*, denoted by *μ*(*X*) = *E*[*Y*|*X*], and for predicting the probability of being treated conditional on *X*, which is commonly referred to as the propensity score, *p*(*X*) = *Pr*(*D* = 1|*X*). The estimates of these so-called plug-in parameters *μ*(*X*) and *p*(*X*) are incorporated into the estimation of treatment effects.

Section 6.1 describes the estimation of individualized treatment effect estimates for every observation in the sample as a function of its covariates *X* (CATEs) based on the causal forest. The estimated parameters obtained from the latter then permit estimating the ATE, as described in Section 6.2. Section 6.3 demonstrates the use of the causal forest estimates for evaluating treatment effects in different customer segments defined by selected covariates. Finally, Section 6.4 shows how to determine which customers should optimally be targeted by the different coupon campaigns to maximize effectiveness in terms of purchases.

### 6.1 Treatment effect heterogeneity

In order to generate the causal forest, the algorithm draws 2,000 random samples that contain 50% of the observations in the data set. In each random sample, it estimates a causal tree as follows: first, a randomly selected subset of covariates is chosen, the number of which is determined by means of cross-validation. The algorithm then uses these covariates for splitting the sample into two subsamples such that the CATEs in the two resulting subsamples are as heterogeneous as possible. More precisely, the algorithm determines both the covariate and the value at which the sample should be split (e.g., age < 25 vs. age ≥ 25) to maximize effect heterogeneity. Intuitively, the algorithm considers all possible splits on values of the considered covariates to find the optimal split in terms of effect heterogeneity. These nodes are split into a larger number of nodes by repeating the splitting procedure until no further splits can be performed without yielding nodes containing less than a certain number of treated and control observations, where the minimum number of observations is determined by cross-validation. The causal forest is finally obtained by averaging over the splitting structure of all 2,000 causal trees.

The CATE in the subsamples resulting from each potential split is estimated by means of an approach proposed by Robinson [98] that allows estimating the CATE with $\sqrt{n}$ consistency. The approach builds on first predicting the plug-in parameters *μ*(*X*) and *p*(*X*), which can be estimated using any predictive ML algorithm with a convergence rate faster than *n*^−1/4^, and then using the predicted plug-in parameters for estimating the CATE. In our case, we estimate the plug-in parameters by means of regression forests with out-of-bag prediction. That is, the method repeatedly draws random subsamples from the original data to learn predictive models for *μ*(*X*) and *p*(*X*), but the predictions for any observation in the data exclusively rely on models from subsamples in which the respective observation is not present. In a second step, the algorithm calculates the residuals $Y_{i,t}-\hat{\mu }\left(X_{i,t}\right)$ and $D_{i,t}-\hat{p}\left(X_{i,t}\right)$ for all observations *i*, *t* in the random sample used for learning the causal tree. In order to determine the split that maximizes effect heterogeneity in the resulting subsamples, the algorithm regresses $Y_{i,t}-\hat{\mu }\left(X_{i,t}\right)$ on $D_{i,t}-\hat{p}\left(X_{i,t}\right)$ in each subsample. That is, for every potential node, the algorithm estimates the following function, where $\hat{\theta }\left(X\right)$ denotes the estimated CATE:

$$\begin{array}{c} Y-\hat{\mu }\left(X\right)=\left(D-\hat{p}\left(X\right)\right)\hat{\theta }\left(X\right). \end{array}$$ (3)

Through averaging over the resulting 2,000 causal trees, the causal forest provides the final estimates of the CATEs $\hat{\theta }\left(X\right)$, i.e., estimations of individualized treatment effects for every point in *X*. To account for the issue that the behavior of one and the same customer is in general not independent across different campaign periods we cluster standard errors at the customer level. The estimation is performed in the statistical software R [99] by means of the causal_forest function provided in the grf package by Tibshirani et al [100].

For the sake of comprehensibility, we have presented a somewhat simplified version of the causal forest algorithm, as provided in the grf package. For one, the causal_forest function does not determine the splitting rules by estimating the CATEs in all possible subsamples. The algorithm, rather, approximates the between-node effect heterogeneity generated through every potential split by means of a gradient for each observation in order to improve computational efficiency. Additionally, the algorithm involves several conditions for formulating splitting rules that aim at avoiding imbalance in the size of the nodes. Explaining these rules in detail would go beyond the scope of this discussion. The manual to the grf package, however, provides all the details (see [101]). Finally, the cross-validation procedure to determine the number of covariates used for building causal trees, the minimum node size as well as different other hyperparameters (see [101] for more details) provided in the grf package relies on a measure of the estimation error suggested by Nie and Wager [102]. This error measure is tailored to the evaluation of treatment effects (rather than predicting outcomes as in predictive ML), but is only one of different possible measures. The algorithm splits the data into two subsamples, then grows causal forests using 100 distinct randomly selected sets of hyperparameters and finally selects the set of hyperparameters with the smallest estimated error. We note that a complication in defining an error measure to be minimized by cross-validation in the context of causal ML is that the true CATEs are not observed. Therefore, the causal forest algorithm cannot compare observed and predicted CATEs for cross-validation purposes, while predictive algorithms can compare predicted and observed outcomes.

### 6.2 Average treatment effect

The estimated causal forest is further used to identify the ATE of coupon provision and thus to assess the overall effectiveness of the coupons (and that of selected coupon types). Athey and Wager [103] propose to estimate the ATE by means of a modified version of the Augmented Inverse Probability Weighting (AIPW) estimator, a doubly robust estimator proposed by Robins et al [104], that is based on weighting the observations by the inverse of their estimated propensity score. This weighting of observations makes the treatment and the control group comparable in terms of their propensity scores and hence the distribution of relevant covariates *X* (for more information on the AIPW estimator, see e.g., [105]). Double robustness is achieved by estimating the ATE via an orthogonalized function, i.e., the predicted plug-in parameters are included in the estimation such that small estimation errors in either predictor result in an overall negligible error and hence do not introduce bias in the estimation of the ATE. The formula used for estimating the ATE is as follows:

$$\begin{array}{ccc} \hat{\Theta } & = & \frac{1}{NT}\sum_{i\in N,t\in T}\Gamma_{i,t}\\ \text{with}\;\Gamma_{i,t} & = & \hat{\theta }\left(X_{i,t}\right)+\frac{D_{i,t}-\hat{p}\left(X_{i,t}\right)}{\hat{p}\left(X_{i,t}\right)\left(1-\hat{p}\left(X_{i,t}\right)\right)}(Y_{i,t}-\hat{\mu }\left(X_{i,t}\right)-(D_{i,t}-\hat{p}\left(X_{i,t}\right))\hat{\theta }\left(X_{i,t}\right)) \end{array}$$ (4)

where the estimates of the plug-in parameters $\hat{\theta }\left(X\right),\hat{p}\left(X\right)$ and $\hat{\mu }\left(X\right)$ for the doubly robust score Γ_*i*,*t*_ are obtained from the causal forest. As mentioned above, $\hat{p}\left(X\right)$ and $\hat{\mu }\left(X\right)$ are predicted by means of regression forests with out-of-bag prediction while $\hat{\theta }\left(X\right)$ is determined using honest trees, i.e., the plug-in estimators for observation (*i*, *t*) are computed based on models learnt in samples that do not contain observation (*i*, *t*). This makes the AIPW-based ATE estimator robust to regularization bias. Thus, similarly to how the CATE is estimated for building causal trees, the modified AIPW estimator by Athey and Wager [103] combines orthogonalization and out-of-sample prediction in order to address the two sources of bias, overfitting and regularization.

A look at Eq (4) reveals that values of $\hat{p}\left(X_{i,t}\right)$ that are either close to zero or close to one can yield large weights for the respective observations, resulting in unstable performance of the estimator. This issue is commonly addressed by trimming the data set, i.e., discarding observations with an estimated propensity score that is below or above certain values. A commonly used trimming rule is to remove observations with estimated propensity scores larger than 0.99 or smaller than 0.01, an approach we also employ in this study.

In our application, we estimate the ATE using the average_treatment_effect function provided in the grf package for R by Tibshirani et al [100], with standard errors clustered at the customer level.

### 6.3 Group average treatment effects

In order to assess the impact of coupon provision across different predefined customer groups, we also estimate so-called Group Average Treatment Effects (GATEs), which correspond to the ATEs in subgroups defined upon selected covariates. The covariates used to define these subgroups are the (original) age group and family size variables, a variable for average daily expenditures that divides the sample into four subgroups of similar size, and a variable measuring income in broader categories, each of which combines two of the more fine-grained income groups in the original variable. We estimate the GATEs based on linear regressions of the doubly robust scores ${\hat{\Gamma }}_{i,t}$ (see Eq (4)) on these categorical variables that indicate which subgroup a customer belongs to, see [106] for more details. To this end, we apply the best_linear_projection function provided in the grf-package.

### 6.4 Optimal policy learning

The optimal policy learning approach by Athey and Wager [10] goes one step further, in the sense that it does not only estimate the effect of coupon provision in predefined customer groups. Rather, it exploits the heterogeneity in coupon effects to determine the coupon distribution rule that maximizes the overall effect of the coupon campaign. Based on observed covariates, the coupon distribution rule distinguishes customer segments that are likely to increase their purchasing behavior upon receiving a coupon from those customer groups not anticipated to respond positively to the campaign. More formally, the algorithm considers specific decision (or policy) rules for whether a coupon should be offered to a customer as a function of the covariate values in *X*, e.g., the customer’s age. Let us denote by *π*(*X*) such a decision rule, which could, for instance, impose that only elderly, but not younger, customers obtain a coupon.

Mathematically speaking, the rule maps a customer’s observed characteristics to the binary treatment decision of whether or not to target the customer through the coupon campaign: *π*: *X* → 0, 1. Optimal policy learning consists of learning the optimal rule among an assumably limited set of implementable candidate policies, where we use Π to denote this set. For instance, another possible rule of how to distribute coupons (in addition to the age-based rule) could be to offer them only to customers with a high volume of previous purchases. Then, both the age- and purchase-dependent rule would enter the set of feasible coupon policies provided in Π.

For learning the optimal coupon policy, the algorithm of Athey and Wager [10] makes use of the doubly robust scores ${\hat{\Gamma }}_{i,t}$ (see Eq (4)). These individual- and time-specific treatment effect estimates are plugged into the following objective function, which aims at maximizing the effectiveness of the coupon campaign by selecting the policy rule with the highest average effect among all policies *π* that are available in the set Π:

$$\begin{array}{c} \pi^{*}=argmax\{\frac{1}{NT}\sum_{i\in 1,...,N}\sum_{t\in 1,...,T}^{}\left(2\pi \left(X_{i,t}\right)-1\right){\hat{\Gamma }}_{i,t}:\pi \in \Pi \} \end{array}$$ (5)

The optimal policy learning approach does not require defining the policies to be considered a priori, but only the number of customer segments between which coupon allocation can differ and the set of covariates that can be considered for determining these customer segments. Thus, the approach identifies the optimal coupon policy in a data-driven way. To determine the optimal coupon distribution strategy, i.e., the one that maximizes the objective function in 5, the algorithm applies a tree-based approach that considers all possible covariate-defined sample splits for generating the customer segmentation (according to the pre-defined number of segments) and all possible coupon assignment strategies within these segments. The resulting coupon distribution rule can be represented as a decision (or policy) tree, i.e., a tree-shaped graph indicating at which values of which covariate the sample is split and which of the resulting customer segments shall receive coupons.

We estimate decision trees of depth 3, implying that we distinguish 8 customer segments for defining the optimal distribution of coupons by means of the policytree package for R by Sverdrup et al [107]. For determining the customer segments, we use all the customer characteristics available in the data set, i.e., age and income group, family size, marital status, and dwelling type. We redefine these variables by setting all missing values to -1, which allows us to omit the variables that indicate missing observations. Then, we also include the customers’ pre-campaign purchasing behavior. Since the algorithm performs a sample split at every possible value of each covariate, i.e., at each observed value, continuous variables can cause performance issues by driving up the number of sample splits. We, therefore, round the pre-campaign average daily expenditures to round values, namely to the nearest 100 for values between 0 and 1,000 and to the nearest 200 for values between 1,000 and 2,000. Further, we group all 157 observations with average daily expenditures of 2,000 or more into one category and include dummies that indicate whether a customer purchased items from the different product categories in the period prior to the campaign. This way, we still capture pre-campaign differences in purchasing behavior well, while substantially reducing the number of sample splits that need to be performed.

### 6.5 Robustness checks and goodness of fit assessment

As described in Section 4, our data set contains a large number of observations with missing socio-economic information. To investigate the robustness of our results with respect to these missing values, we perform the entire analysis on a reduced data set containing only observations of customers whose socio-economic background is known, i.e., on a data set with 13,792 observations of the purchasing behavior of *n* = 431 individuals.

In a second robustness check, we rerun our estimation of the ATE of receiving coupons (of a certain type) in the full sample, but using an alternative causal ML approach, namely Double ML [64]. Just as the causal forest approach, Double ML relies on combining effect estimation based on Neyman [75]-orthogonal scores with sample splitting. For estimating the plug-in parameters, we use Lasso regression with 10-fold cross-validation after augmenting the set of covariates with interaction and second order terms. The estimation is performed in the statistical software R [99] by means of the causalDML function provided in the causalDML package by Knaus [108] (see the manual to the causalDML package for more details).

Finally, we also assess how well the causal forest approach captures the total effect of coupon reception as well as effect heterogeneity. The fact that the true CATEs and ATE are unobservable prevents us from relying on performance measures that are commonly used to evaluate the performance of ML algorithms for predictive purposes. We therefore investigate the goodness of fit of the causal forest by means of the test_calibration function included in the grf package. Put simply, this function regresses the estimated CATEs on the average of all CATEs as estimated by the causal forest as well as on the difference between the CATE estimate for the respective observation and the average CATE estimate. A coefficient close to 1 on the average of all forest predictions suggests that the mean forest estimate for the CATEs is correct. A coefficient close to 1 for the difference between the individual CATE estimate and the average over all estimates indicates that the heterogeneity estimates from the forest are well calibrated. Further, this coefficient informs about the presence of heterogeneity. If it is significantly larger than 0, we can reject the null hypothesis of no heterogeneity.

## 7 Empirical results

### 7.1 Treatment effect heterogeneity

**Fig 1:**
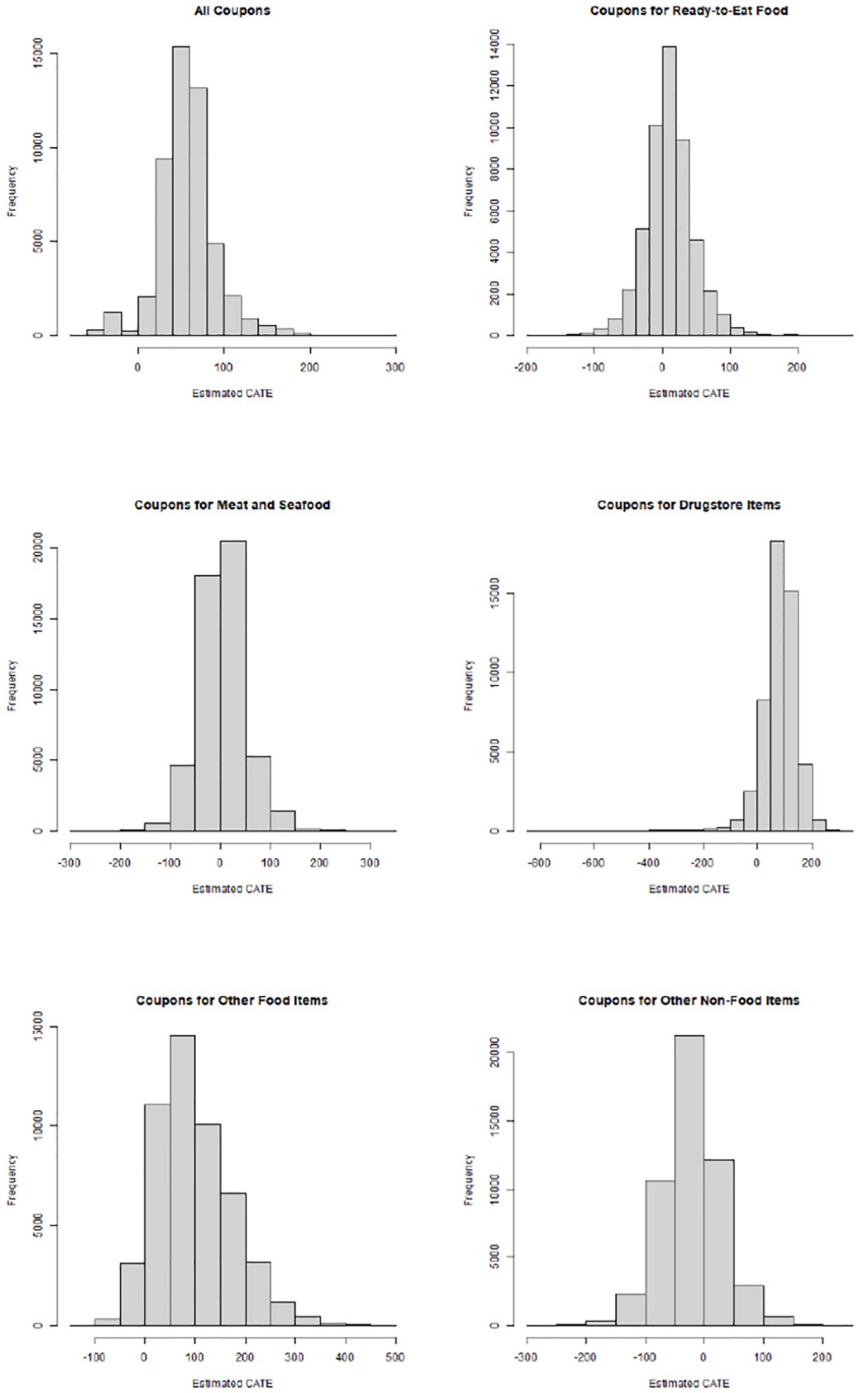
Distribution of CATE by coupon type. Distribution of CATE estimates for receiving any coupon, as well as coupons applicable to ready-to-eat food, meat and seafood, other food, drugstore products and other non-food products, denoted in monetary units.

Fig 1 shows the distribution of the individualized treatment effects (CATEs) as estimated by means of the causal forest algorithm outlined in Section 6.1. The treatment effect of being provided with any coupon is positive for the vast majority of observations and, except for some outliers, ranges between -100 and 200 monetary units. Similarly, the provision of drugstore coupons and coupons applicable to other food have a positive effect for the majority of observations. The distributions of the effects of coupons applicable to ready-to-eat food as well as meat and seafood, however, seem to be rather centered around zero, such that the estimates are positive and negative, respectively, for about half of the observations. For coupons applicable to other non-food products, we even find a negative effect on daily expenditures for the majority of observations. The plots suggest greater heterogeneity in the treatment effects of the individual coupon categories than when pooling all coupons. It appears that the effects of the different coupon categories cancel each other out to some extent when combined in one analysis, implying that the different coupon categories should best be analyzed separately.

The heterogeneities in CATEs as revealed by the causal forest approach suggest not just assessing the ATE, as in Section 7.2, but also investigating how the effect of coupons (of certain categories) differs between customer groups defined by covariates *X*, see Section 7.3. Finally, heterogeneous effects motivate our aim to learn the optimal coupon distribution scheme across customers that maximizes the expected ATE of coupon provision, see Section 7.4.

### 7.2 The causal effect of receiving coupons

Table 2 shows the estimated ATE of receiving any coupon on daily expenditures in the campaign period, as well as that of receiving coupons from each of the five coupon categories, based on the AIPW approach outlined in Section 6.2. The results suggest that receiving any coupon has a positive and statistically significant effect on daily expenditures during the campaign period. Providing a customer with a coupon increases her expected daily expenditures by some 60 monetary units. The effect estimates for the different coupon categories provide a more nuanced picture. Provision of coupons for drugstore items and other food has a statistically significant positive effect on daily spending during the campaign period. Receiving coupons that belong to these categories increases expected average daily expenditures during the validity period by some 80 and 78 monetary units, respectively. Handing out coupons applicable to other non-food products, on the other hand, is estimated to decrease a customer’s expected average daily expenditures by some 24 monetary units, with this result also being statistically significant. The estimated ATE of providing coupons from the other two categories has no statistically significant effect on the customers’ expected daily spending during the campaign period. A possible explanation for the insignificant or significantly negative effect of these latter three coupon types is that the receipt of such coupons may not incentivize people to buy, but that such coupons are mainly used for products that the coupon recipient would have purchased anyway.

**Table 2:** ATE estimates for coupon reception (general/by coupon type).

	Coef.	Standard Error	Sign. Level
*ATE: receiving any coupon*	59.56	5.554	***
*ATE: receiving coupon for ready-to-eat food*	4.49	7.414	
*ATE: receiving coupon for meat/seafood*	6.43	7.174	
*ATE: receiving coupon for other food*	78.48	12.071	***
*ATE: receiving coupon for drugstore items*	80.19	6.223	***
*ATE: receiving coupon for other non-food items*	-23.63	6.678	***

ATE of receiving any coupon as well as the ATEs of receiving coupons applicable to specific product categories, each with standard error and significance level. The estimates are denoted in monetary units. Significance levels:

. p<0.1,

* p<0.05,

** p<0.01,

*** p<0.001.

As discussed in Section 5.2, coupon provision may, on the one hand, have longer-term positive effects on purchasing behavior by increasing customer loyalty, and on the other hand, bring about inter-temporal spillovers by inducing customers to advance their purchases to periods when they have coupons applicable to them. We therefore also take a look at the overall effect of coupon reception in *t* on daily expenditures in the following campaign period (*t* + 1) and the period thereafter (*t* + 2) (see Table 3). The results suggest that the effect of coupon provision on daily expenditures is sustainable, i.e., coupon provision in *t* not only has a short-term effect on purchases in *t*, but also has a statistically significant, albeit smaller, effect on purchases in subsequent periods. This may be due to a coupon-induced increase in customer retention (but also to indirect effects, see the discussion in Section 5.2).

**Table 3:** Longer-term ATE estimates.

	Effect in *t* + 1			Effect in *t* + 2		
	Coef.	Standard Error	Sign. Level	Coef.	Standard Error	Sign. Level
*ATE: receiving any coupon*	38.69	4.156	***	31.18	5.417	***
*ATE: ready-to-eat food coupons*	-27.76	6.618	***	1.18	8.838	
*ATE: meat/seafood coupons*	12.01	6.048	*	5.75	6.337	
*ATE: other food coupons*	51.61	13.499	***	2.88	8.217	
*ATE: drugstore coupons*	101.09	5.10	***	103.73	6.373	***
*ATE: other non-food coupons*	32.78	5.918	***	15.98	5.644	**

ATE on daily expenditures in period after coupon campaign (*t* + 1) and the period thereafter (*t* + 2), each with standard error and significance level. The estimates are denoted in monetary units. Significance levels:

. p<0.1,

* p<0.05,

** p<0.01,

*** p<0.001.

The longer-term effect of drugstore and other food coupons is also positive and statistically significant, with drugstore coupons showing an even larger effect on purchasing behavior in both post-treatment periods than in the short term. Coupons applicable to other non-food products, that in the short run have a statistically significant negative effect, show a statistically significant positive effect on daily spending in the subsequent periods. One possible explanation for this finding is that, while in the short run these coupons were only redeemed for the purchase of products that would have also been purchased without the coupons, in the longer term they may have increased customer loyalty.

The estimated effect of meat and seafood coupons on expenditures in *t* + 1 and *t* + 2 is not statistically significant, while that of ready-to-eat food coupons is even significantly negative for the outcome in *t* + 1, which may indicate spillover effects that are not offset by positive expenditure-increasing effects. For ready-to-eat food and meat/seafood coupons, we can therefore conclude that they do not seem to be an effective marketing tool for increasing customer spending, neither in the short nor in the longer run.

In the following, we will again focus on the short-term effect of coupon provision in period *t* on daily expenditures in period *t*. The next section examines effect heterogeneity with regard to selected customer characteristics. This is because the provision of coupons could significantly increase spending of certain customer groups, despite not having a statistically significant effect on the overall customer base. Similarly, providing coupons applicable to drugstore or other food could have a significant impact on purchasing behavior only among certain subgroups of customers.

### 7.3 Group average treatment effects

In this section, we assess how the provision of coupons affects different customer groups, by estimating Group Average Treatment Effects (GATEs) as discussed in Section 6.3. We investigate how the effect of providing coupons differs depending on customers’ age, income, family size and pre-campaign expenditures. Furthermore, we also examine the GATEs of those coupon categories with a highly statistically significant ATE, i.e., drugstore coupons and coupons applicable to other food.

**Fig 2:**
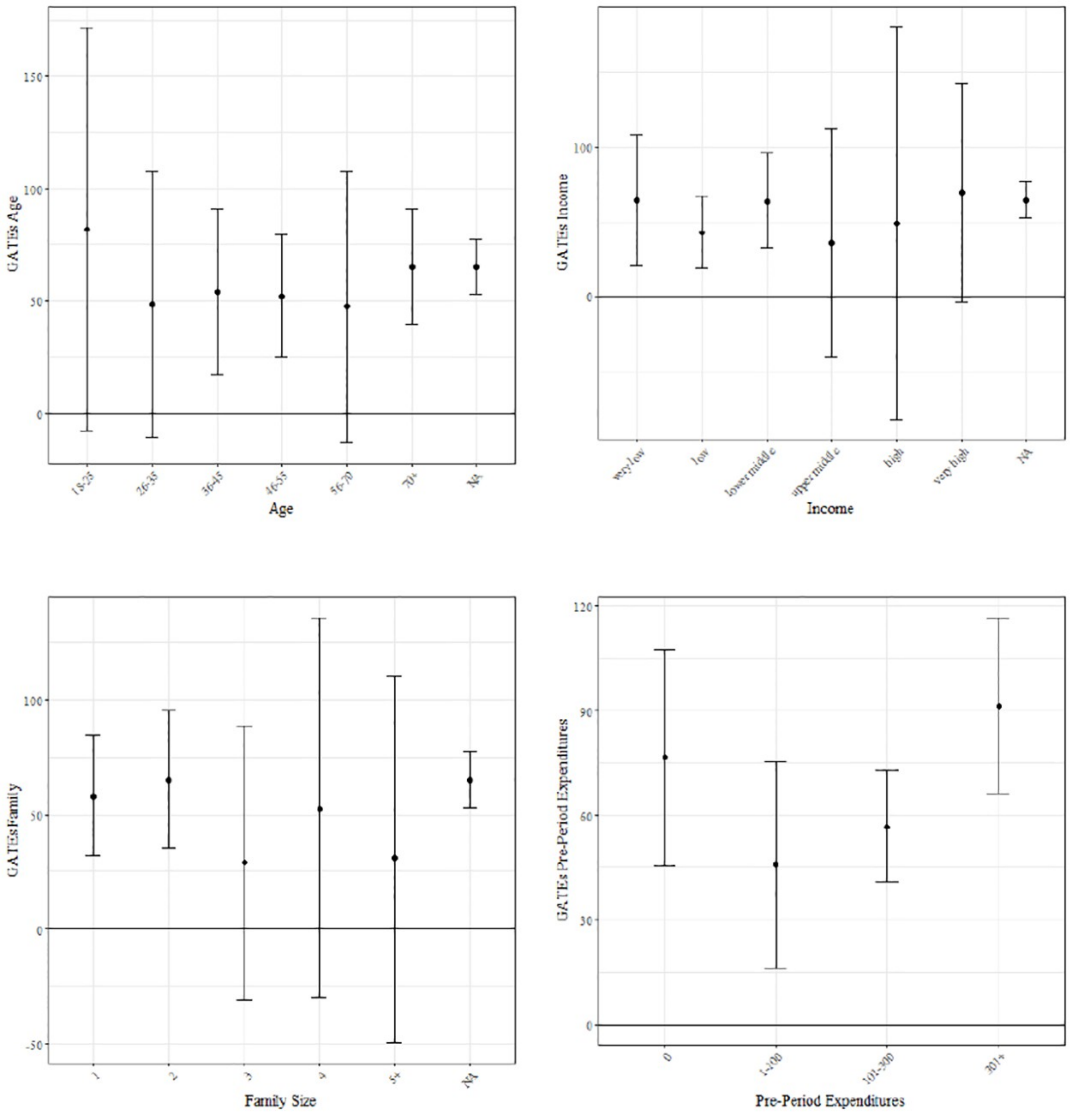
GATE estimates of receiving any coupon. GATE estimates of receiving any coupon by age, income, family size and pre-campaign expenditures with 95% confidence interval, denoted in monetary units.

Fig 2 shows the GATEs of receiving any coupon by age, income, family size and pre-campaign expenditures, respectively. The graphs suggests that providing coupons has a positive effect on purchasing behavior in every customer group and most effect estimates are statistically significant. The effect appears to be particularly large among customers from smaller households and among those who made either no or large purchases in the period prior to the campaign.

**Fig 3:**
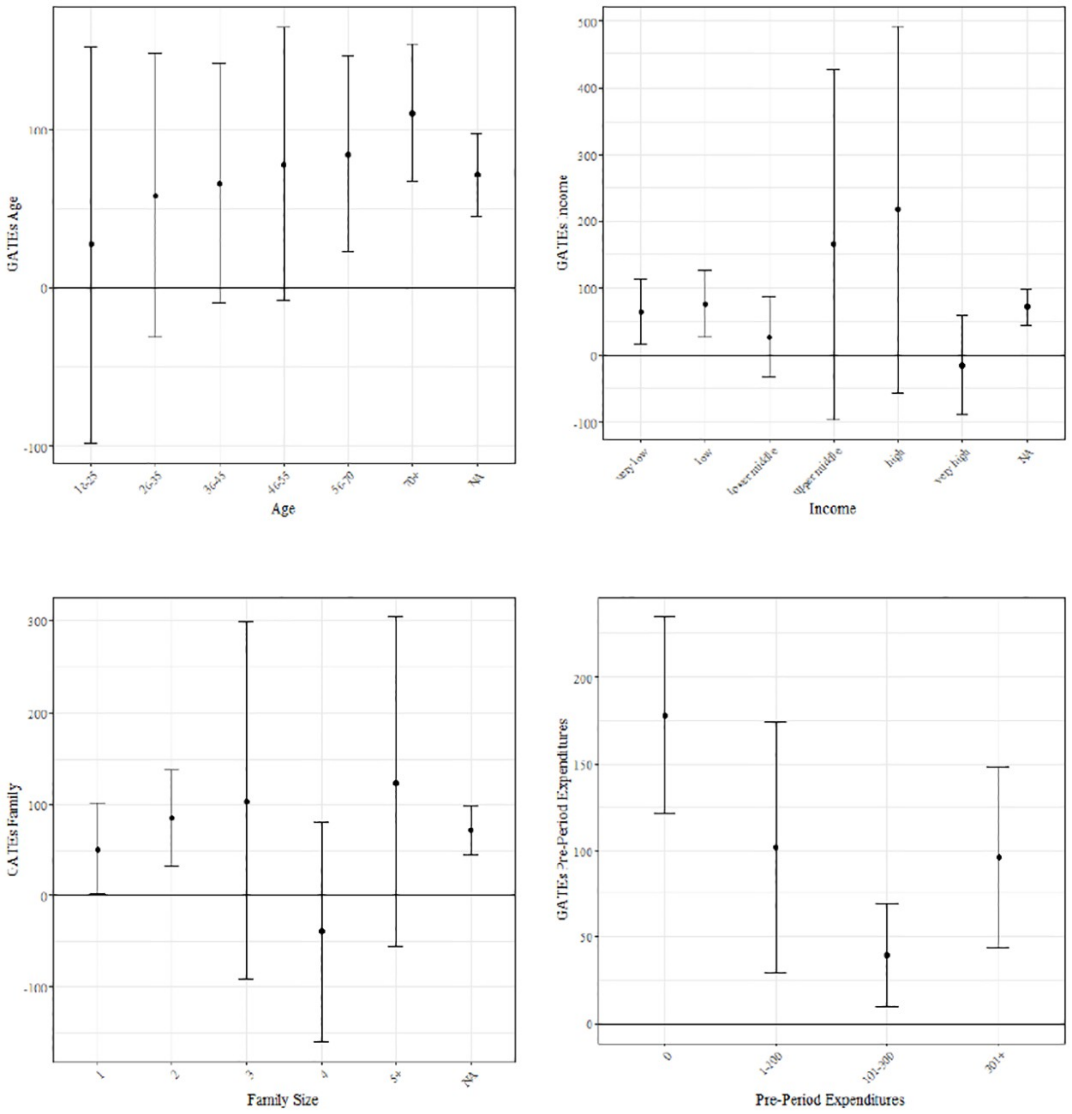
GATE estimates of coupons applicable to other food. GATE estimates of coupons applicable to other food items with 95% confidence interval, denoted in monetary units.

The GATE charts in Fig 3 show that in almost all subgroups considered, the provision of coupons for other food has a positive effect on daily spending, which is in many cases statistically significant. The most pronounced differences in GATEs can be found among customer subgroups defined by average daily spending prior to the campaign period. The effect of food coupons tends to be high and statistically significant for previously inactive customers, while it is much smaller, though still statistically significant, for customers with high pre-period spending. This may suggest that coupons for other food have the potential to reactivate dormant customers. This hypothesis is also supported by the fact that the provision of coupons applicable to other food has a relatively large statistically significant effect among customers for whom information on socio-economic characteristics is not available. Customers for whom no information is available may be more likely to have low loyalty to the store and to be rather inactive, but they may be reactivated by providing them with other food coupons. These results suggest that coupons applicable to other food are efficient for inactive and non-frequent customers, while they have less impact on the purchasing behavior of frequent shoppers.

**Fig 4:**
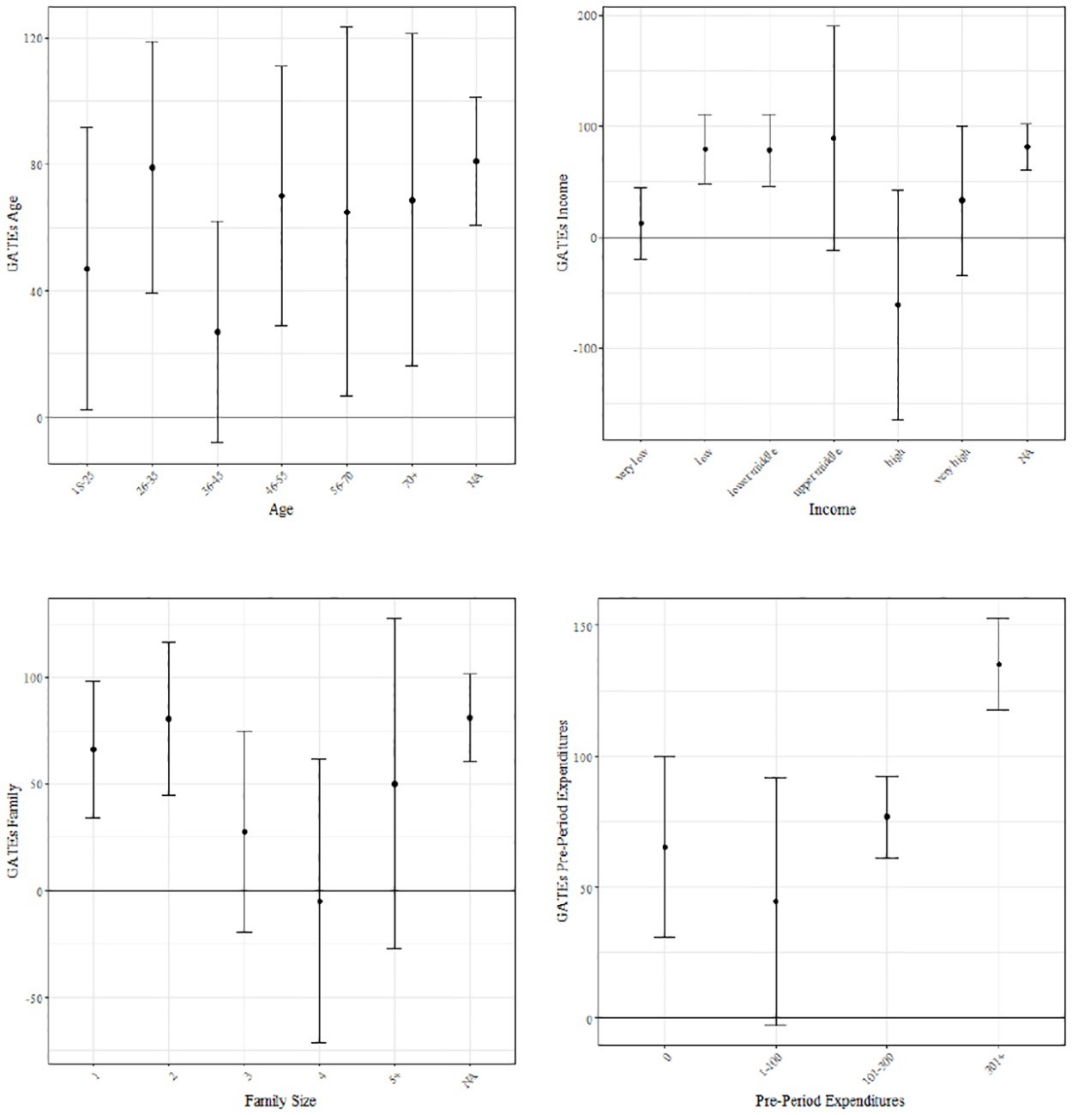
GATE estimates of drugstore coupons. GATE estimates of drugstore coupons with 95% confidence interval, denoted in monetary units.

Fig 4 shows that providing drugstore coupons has a positive effect on daily spending for almost all subgroups considered and that the effect is statistically significant in most cases. Again, the largest difference can be found in the GATE estimates by pre-campaign spending. The effect of drugstore coupons on average per-day expenditures is larger the higher the customer’s pre-campaign spending, which is the reverse pattern of what we find for coupons applicable to other food. This suggests that other food coupons are more efficient at reactivating dormant customers and drugstore coupons at retaining frequent shoppers. S4 Fig shows the GATE plots for ready-to-eat food coupons, S5 Fig those for meat/seafood coupons, and S6 Fig those for coupons applicable to other non food products.

### 7.4 The optimal distribution of coupons

While the ML-based estimation of ATEs and GATEs are very useful tools for evaluating the average effects of coupon campaigns (across subgroups), it is not necessarily most appropriate for optimizing strategies for later coupon campaigns. For the latter task, we apply the optimal policy learning framework by Athey and Wager [10] to determine which customer groups should be provided with coupons in order to maximize their average impact.

**Fig 5:**
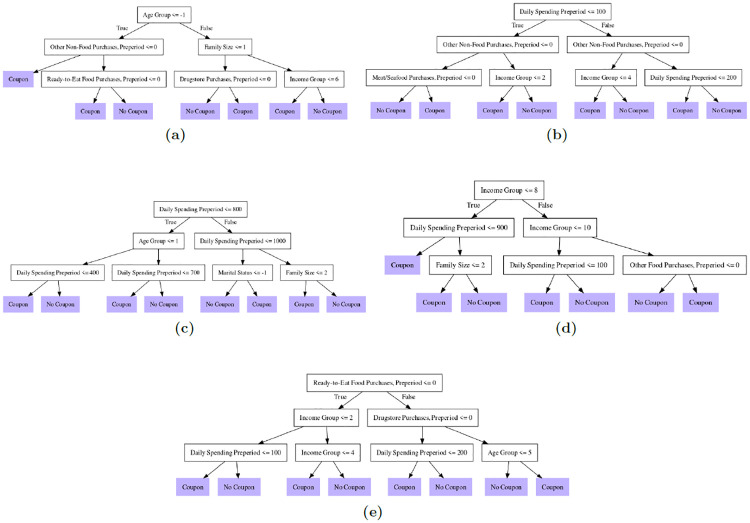
Depth-3 trees for optimal coupon provision, by coupon type. Depth-3 trees for optimal provision of coupons applicable to (a) ready-to-eat food, (b) meat and seafood, (c) other food, (d) drugstore products as well as (e) other non-food products.

Fig 5 shows the optimal distribution rules (or policies) for each coupon category as suggested by the optimal policy tree outlined in Section 6.4. The optimal rule for ready-to-eat food coupons (decision tree (a)) suggests providing ready-to-eat food coupons to customers with no drugstore purchases in the pre-campaign period if their marital status is unknown (a value of -1 for the variables family size, marital status, age group and income group implies missingness, see Section 6.4) and their age is unknown or they are not older than 26, or if their marital status is known and they live in a household with at most three members. The retailer should further provide ready-to-eat food coupons to customers who purchased drugstore products in the pre-period if their income is in one of the lowest four income groups or unknown, or if their age is unknown and their average daily purchases in the pre-period were less than 50 monetary units (as daily expenditures are rounded for creating policy trees, all customers with daily expenditures below 50 monetary units fulfil the condition ‘Daily Expenditure Preperiod < = 0’).

The optimal distribution rule for drugstore coupons (decision tree (d)) proposes providing drugstore coupons to those customers with unknown, low, or middle incomes if their daily pre-campaign expenditures did not exceed 900 monetary units and/or their family does not have more than 2 members. Customers belonging to the higher-middle income group should receive drugstore coupons if their average in-store spending did not exceed 100 monetary units per day in the period before the campaign. Customers belonging to the high-income group, finally, should only be provided with drugstore coupons if they purchased other food products at the store in the pre-campaign period.

The distribution rules paint a similar picture as the GATE estimates in Section 7.1 about which customer groups are likely to be positively impacted by the provision of certain coupon types. In contrast to the effect heterogeneity analysis across pre-specified subgroups in Section 7.1, the optimal policy tree determines the covariate values at which the sample should optimally be split as well as the groups of coupon recipients and non-recipients in a data-driven way, in order to maximize the average effect of the campaign.

The other decision trees can be interpreted accordingly. A look at the covariates used for sample splitting in those other decision trees shows that each observed customer characteristic is used for defining the distribution rules of at least one coupon type.

### 7.5 Robustness checks and goodness of fit assessment

The ATEs based on the reduced data set containing only observations of customers whose socio-economic background is known are presented in Table 4. They are close to the ATE estimates in the full data set, although the standard errors are considerably larger, due to the much smaller number of observations.

**Table 4:** ATE of coupon reception (general/by coupon type), estimated in reduced data set.

	Coef.	Standard Error	Sign. Level
*ATE: receiving any coupon*	61.63	11.969	***
*ATE: receiving coupon for ready-to-eat food*	-2.26	10.282	
*ATE: receiving coupon for meat and seafood*	-31.74	10.095	**
*ATE: receiving coupon for other food*	133.61	46.159	**
*ATE: receiving coupon for drugstore items*	72.70	9.272	***
*ATE: receiving coupon for other non-food items*	-21.30	9.721	*

ATE of receiving any coupon as well as the ATEs of receiving coupons applicable to specific product categories in the reduced data set (without observations with missing socio-economic information), each with standard error and significance level. The estimates are denoted in monetary units. Significance levels:

. p<0.1,

* p<0.05,

** p<0.01,

*** p<0.001.

The GATE and policy tree plots are provided in the Supporting information Section (see S7–S12 Figs for the GATE estimates of receiving any coupon, ready-to-eat food coupons, meat/seafood coupons, other food coupons, drugstore coupons and other non-food coupons, respectively. The policy tree plots are provided in S13 Fig). The GATE plots show similar patterns in how different customer groups are affected by each coupon type, although they are not exactly identical with the GATEs estimated in the full sample. In the policy tree plots, the splitting rules are based on a similar set of variables with similar cutting points as those estimated in the full data set. These results suggest that the large number of observations with missing socio-economic information does not introduce systematic bias into the estimation of the treatment effects and the optimal coupon distribution scheme. The longer-term ATE estimates are provided in Table 5.

**Table 5:** Longer-term ATE estimates, estimated in reduced data set.

	Effect in *t* + 1			Effect in *t* + 2		
	Coef.	Standard Error	Sign. Level	Coef.	Standard Error	Sign. Level
*ATE: receiving any coupon*	35.97	8.94	***	29.47	9.166	**
*ATE: ready-to-eat food coupons*	-11.96	9.642		-21.43	10.344	*
*ATE: meat/seafood coupons*	-3.94	9.338		-14.71	8.962	
*ATE: other food coupons*	41.33	25.738		36.99	27.199	
*ATE: drugstore coupons*	96.91	8.615	***	62.99	13.487	***
*ATE: other non-food coupons*	15.98	7.05	*	5.58	9.893	

ATE on daily expenditures in period after each coupon campaign (*t* + 1) and the period thereafter (*t* + 2), estimated in the reduced data set (without observations with missing socio-economic information), each with standard error and significance level. The estimates are denoted in monetary units. Significance levels:

. p<0.1,

* p<0.05,

** p<0.01,

*** p<0.001.

The (short-term) ATE estimates obtained from Double ML with Lasso regression are provided in Table 6 and the longer-term Double ML-based ATE estimates in Table 7. They are similar to the causal forest estimates, indicating that the results are robust to the ML algorithm used for predicting the plug-in parameters.

**Table 6:** ATE of coupon reception estimated by means of double ML.

	Coef.	Standard Error	Sign. Level
*ATE: Receiving any coupon*	49.37	4.573	***
*ATE: Receiving coupon for ready-to-eat food*	27.24	27.82	
*ATE: Receiving coupon for meat/seafood*	16.38	10.762	
*ATE: Receiving coupon for other food items*	49.28	13.854	***
*ATE: Receiving coupon for drugstore items*	50.04	4.449	***
*ATE: Receiving coupon for other non-food items*	6.50	26.254	

ATE of receiving any coupon as well as the ATEs of receiving coupons applicable to specific product categories estimated by means of Double ML, each with standard error and significance level. The estimates are denoted in monetary units. Significance levels:

. p<0.1,

* p<0.05,

** p<0.01,

*** p<0.001.

**Table 7:** Longer-term ATE estimates, estimated by means of double ML.

	Effect in *t* + 1			Effect in *t* + 2		
	Coef.	Standard Error	Sign. Level	Coef.	Standard Error	Sign. Level
*ATE: Receiving any coupon*	34.89	3.933	***	29.67	4.783	***
*ATE: ready-to-eat food coupons*	-10.29	21.85		34.55	17.155	*
*ATE: meat/seafood coupons*	22.21	8.812	*	11.62	5.109	*
*ATE: other food coupons*	-12.66	28.303		9.71	3.876	*
*ATE: drugstore coupons*	282.94	222.941		17.44	3.992	***
*ATE: other non-food coupons*	41.12	8.328	***	53.28	3.361	***

ATE on daily expenditures in period after each coupon campaign (*t* + 1) and the period thereafter (*t* + 2), estimated by means of Double ML, each with standard error and significance level. The estimates are denoted in monetary units. Significance levels:

. p<0.1,

* p<0.05,

** p<0.01,

*** p<0.001.

Finally, Table 8 provides the results for the goodness-of-fit tests. The latter do not point to a misspecification of the CATE models of receiving any coupons, coupons for other food items, drugstore coupons and coupons for other non-food items. Furthermore, they indicate that the causal forests for receiving any coupon and drugstore coupons appropriately capture effect heterogeneity. For other food coupons, the other coupon category that showed a statistically significant positive effect on sales, however, we cannot reject the null hypothesis that there is no effect heterogeneity. For the categories of ready-to-eat food and meat/seafood coupons, however, the tests do not suggest that the mean forest estimates are correct or that the causal forests appropriately capture effect heterogeneity. We recall that for these coupons, we did not find any significant effects on customer spending.

**Table 8:** Goodness-of-fit test results.

	Mean Estimate	Standard Error	Sign. Level	Differential	Standard Error	Sign. Level
*Any coupon*	1.01	0.13	***	0.74	0.15	***
*Ready-to-eat food coupons*	3.16	1.78	*	-1.21	0.604	
*Meat/seafood coupons*	3.67	3.726		-0.53	0.467	
*Other food coupons*	0.85	0.238	***	-0.36	0.395	
*Drugstore coupons*	1.05	0.257	***	0.95	0.406	**
*Other non-food coupons*	1.11	0.783	.	0.01	0.335	

Results for the goodness-of-fit test. Significance levels:

^.^ p<0.1,

* p<0.05,

** p<0.01,

*** p<0.001.

## 8 Conclusion

In this paper, we presented different causal ML methods and applied them to evaluate a marketing intervention, namely a coupon campaign in a retail store. We assessed the average effects of coupons for different product categories, as well as the extent to which the effects differed across predefined subgroups of customers, for instance, between clients with relatively high vs. low prior purchases. Finally, we determined in a data-driven way which customer segments should optimally be provided with a coupon to maximize effectiveness in terms of sales, a method known as optimal policy learning.

We found that coupons for only two out of five product categories, namely ‘drugstore articles’ and ‘other food’, had a positive and statistically significant overall effect on purchases, while receiving coupons for other non-food products actually significantly reduced customers’ daily spending. Additionally, we detected several customer segments whose purchasing behavior was influenced particularly strongly by certain types of coupons and should therefore be targeted by coupon campaigns to maximize the effect on sales. Such information appears very useful for retailers to optimally design marketing campaigns with regard to their impact.

Causal ML methods can be applied to evaluate and optimize business strategies also in other domains than the one considered in this paper, if sufficiently rich observational or experimental data on treatments (like business decisions or firm policies), outcomes (like key performance indicators or sales), and covariates (like customer characteristics) are available. Further potential applications for causal ML are the evaluation and optimization of online marketing interventions (like online ads), loyalty programs and other campaigns for tackling customer attrition and churn, but also the assessment of different employee benefit plans and compensation schemes, designs of job postings, or in-house training programs, among many other potential use cases.

## Supporting information

S1 Table Daily expenditures by product type. Mean of daily expenditures by brand and product type in the total sample (’Overall‘), among coupon receivers and non-receivers as well as the mean difference across treatment states and the p-value of a two-sample t-test.
(PDF)

S2 Table Descriptive statistics, variable means among (non-)recipients of different coupon types. Mean of the variables among the treated who received a coupon of a certain category and of those who did not. The coupons of each category are applicable to the following product categories defined by the retailer: (a) ready-to-eat food coupons: ‘Bakery’, ‘Restaurant’, ‘Prepared Food’, ‘Dairy, Juices & Snacks’, (b) meat and seafood coupons: ‘Meat’, ‘Packaged Meat’, ‘Seafood’, (c) coupons applicable to other food: ‘Grocery’, ‘Salads’, ‘Vegetables (cut)’, ‘Natural Products’, (d) drugstore coupons: ‘Pharmaceutical’, ‘Skin & Hair Care’, and (e) coupons applicable to other non-food products: ‘Flowers & Plants’, ‘Garden’, ‘Travel’, ‘Miscellaneous’.
(PDF)

S1 Fig Propensity scores of receiving any coupon & ready-to-eat food coupons. Distribution of propensity scores of receiving any coupon among observations that received (a) no coupon and (b) any coupon, as well as that of the propensity scores of receiving ready-to-eat food coupons among observations that (c) did not and (d) did receive ready-to-eat food coupons. The plots are produced with the logspline command in R with the lower and upper bounds of the support of the propensity scores are set to 0 and 1.
(TIF)

S2 Fig Propensity scores of receiving meat/seafood coupon & other food coupons. Distribution of propensity scores of receiving meat/seafood coupons among observations that (a) did not and (b) did receive meat/seafood coupons, as well as that of the propensity scores of receiving other food coupons among observations that (c) did not and (d) did receive other food coupons. The plots are produced with the logspline command in R with the lower and upper bounds of the support of the propensity scores are set to 0 and 1.
(TIF)

S3 Fig Propensity scores of receiving drugstore coupons & other non-food coupons. Distribution of propensity scores of receiving drugstore coupons among observations that (a) did not and (b) did receive drugstore coupons, as well as that of the propensity scores of receiving other non-food coupons among observations that (c) did not and (d) did receive other non-food coupons. The plots are produced with the logspline command in R with the lower and upper bounds of the support of the propensity scores are set to 0 and 1.
(TIF)

S4 Fig GATEs of ready-to-eat food coupons. GATE estimates of ready-to-eat food coupons with 95% confidence interval, denoted in monetary units.
(TIF)

S5 Fig GATEs of meat/seafood coupons. GATE estimates of meat/seafood coupons with 95% confidence interval, denoted in monetary units.
(TIF)

S6 Fig GATEs of non-food coupons. GATE estimates of coupons applicable to other non-food products with 95% confidence interval, denoted in monetary units.
(TIF)

S7 Fig GATEs of receiving any coupon, estimated in reduced data set. GATE estimates of receiving any coupon with 95% confidence interval, estimated in reduced data set and denoted in monetary units.
(TIF)

S8 Fig GATEs of ready-to-eat food coupons, estimated in reduced data set. GATE estimates of ready-to-eat food coupons with 95% confidence interval, estimated in reduced data set and denoted in monetary units.
(TIF)

S9 Fig GATEs of meat/seafood coupons, estimated in reduced data set. GATEs of meat and seafood coupons with 95% confidence interval, estimated in reduced data set and denoted in monetary units.
(TIF)

S10 Fig GATEs of other food coupons, estimated in reduced data set. GATE estimates of coupons applicable to other food items with 95% confidence interval, estimated in reduced data set and denoted in monetary units.
(TIF)

S11 Fig GATEs of drugstore coupons, estimated in reduced data set. GATE estimates of drugstore coupons with 95% confidence interval, estimated in reduced data set and denoted in monetary units.
(TIF)

S12 Fig GATEs of other non-food coupons, estimated in reduced data set. GATE estimates of coupons applicable to other non-food items with 95% confidence interval, estimated in reduced data set and denoted in monetary units.
(TIF)

S13 Fig Depth-3 trees for optimal coupon provision, estimated in reduced data set. Depth-3 trees for optimally distributing coupons applicable to (a) ready-to-eat food, (b) meat and seafood, (c) other food, (d) drugstore products and (e) other non-food products, estimated in reduced data set.
(TIF)
