# Supplementary material for: How causal machine learning can leverage marketing strategies: Assessing and improving the performance of a coupon campaign
Source: PLoS One. 2023 Jan 11;18(1):e0278937. doi: 10.1371/journal.pone.0278937 (PMC9833560; doi:10.1371/journal.pone.0278937)
Supplement: S1 Table — Mean of daily expenditures by brand and product type in the total sample (’Overall‘), among coupon receivers and non-receivers as well as the mean difference across treatment states and the p-value of a two-sample t-test. (PDF) [file pone.0278937.s001.pdf]

	Overall N = 50,624	Coupon Receivers N = 15,327	Non-Receivers N = 35,297	Diff	p-val
by brand type: established brands	221	255	206	48	0
local brands	59.17	74.01	52.73	21.28	0
by product type: alcohol	0.657	0.975	0.519	0.46	0
bakery	78.79	85.1	76.05	9.05	0
dairy, juices & snacks	4.72	6.08	4.13	1.96	0
flowers & plants	0.699	0.846	0.635	0.21	0.02
fuel	94.35	107.2	88.77	18.42	0
garden	1.93	2.61	1.63	0.98	0
grocery	114	136	105	31	0
meat	82.04	88.45	79.25	9.2	0
miscellaneous	4.7	5.47	4.37	1.1	0
natural products	6	7.11	5.51	1.6	0
packaged meat	87.86	95.4	84.59	10.81	0
pharmaceutical	31.8	38.88	28.72	10.16	0
prepared food	2.48	3.05	2.24	0.81	0
restaurant	76.08	81.94	73.54	8.41	0
salads	1.71	2.16	1.52	0.64	0
seafood	1.86	2.08	1.76	0.32	0.01
skin & hair care	76.94	82.95	74.32	8.63	0
travel	1.7	2.14	1.5	0.64	0
vegetables (cut)	0.017	0.026	0.014	0.01	0.03
