# Supplementary material for: How causal machine learning can leverage marketing strategies: Assessing and improving the performance of a coupon campaign
Source: PLoS One. 2023 Jan 11;18(1):e0278937. doi: 10.1371/journal.pone.0278937 (PMC9833560; doi:10.1371/journal.pone.0278937)
Supplement: S2 Table — Mean of the variables among the treated who received a coupon of a certain category and of those who did not. The coupons of each category are applicable to the following product categories defined by the retailer: (a) ready-to-eat food coupons: ‘Bakery’, ‘Restaurant’, ‘Prepared Food’, ‘Dairy, Juices & Snacks’, (b) meat and seafood coupons: ‘Meat’, ‘Packaged Meat’, ‘Seafood’, (c) coupons applicable to other food: ‘Grocery’, ‘Salads’, ‘Vegetables (cut)’, ‘Natural Products’, (d) drugstore coupons: ‘Pharmaceutical’, ‘Skin & Hair Care’, and (e) coupons applicable to other non-food products: ‘Flowers & Plants’, ‘Garden’, ‘Travel’, ‘Miscellaneous’. (PDF) [file pone.0278937.s002.pdf]

	Ready-to-Eat Food Coupons		Meat/Seafood Coupons		Other Food Coupons	
	Received	Not Received	Received	Not Received	Received	Not Received
daily expenditures	237	257	234	264.37	241	335
age: 18-25	0.03	0.032	0.029	0.034	0.031	0.033
26-35	0.101	0.103	0.092	0.12	0.099	0.159
36-45	0.14	0.143	0.138	0.147	0.142	0.132
46-55	0.191	0.192	0.191	0.192	0.191	0.208
56-70	0.051	0.036	0.044	0.047	0.046	0.029
70+	0.042	0.034	0.042	0.032	0.04	0.021
unknown	0.444	0.461	0.463	0.427	0.452	0.418
family size: 1	0.174	0.167	0.173	0.169	0.172	0.164
2	0.222	0.199	0.214	0.212	0.215	0.175
3	0.079	0.078	0.077	0.083	0.078	0.092
4	0.036	0.045	0.034	0.05	0.038	0.073
5+	0.044	0.05	0.039	0.06	0.045	0.078
unknown	0.444	0.461	0.463	0.427	0.452	0.418
marital status: married	0.239	0.227	0.227	0.246	0.235	0.207
unmarried	0.082	0.088	0.081	0.09	0.084	0.097
unknown	0.679	0.686	0.691	0.664	0.681	0.696
dwelling type: rented	0.031	0.036	0.029	0.041	0.032	0.06
owned	0.525	0.503	0.507	0.532	0.516	0.521
unknown	0.444	0.461	0.463	0.427	0.452	0.418
income group: 1	0.045	0.038	0.041	0.044	0.043	0.022
2	0.049	0.054	0.049	0.053	0.05	0.057
3	0.052	0.044	0.048	0.05	0.049	0.041
4	0.115	0.11	0.109	0.121	0.111	0.164
5	0.14	0.133	0.138	0.135	0.137	0.14
6	0.059	0.066	0.061	0.063	0.062	0.052
7	0.023	0.023	0.025	0.02	0.023	0.016
8	0.028	0.033	0.025	0.038	0.03	0.038
9	0.024	0.024	0.021	0.029	0.023	0.043
10	0.008	0.003	0.006	0.008	0.006	0.008
11	0.003	0.003	0.003	0.002	0.003	0
12	0.011	0.008	0.01	0.01	0.01	0
unknown	0.444	0.461	0.463	0.427	0.452	0.418
coupons redeemed	0.037	0.018	0.036	0.018	0.031	0.006

	Drugstore Coupons		Other Non-Food Coupons	
	Received	Not Received	Received	Not Received
daily expenditures	243	272	246	243.54
age: 18-25	0.031	0.033	0.036	0.028
26-35	0.104	0.07	0.107	0.099
36-45	0.143	0.117	0.149	0.137
46-55	0.191	0.198	0.2	0.187
56-70	0.046	0.035	0.047	0.044
70+	0.039	0.033	0.04	0.038
unknown	0.447	0.514	0.422	0.466
family size: 1	0.172	0.162	0.177	0.168
2	0.215	0.176	0.224	0.207
3	0.079	0.076	0.079	0.079
4	0.04	0.031	0.043	0.038
5+	0.047	0.04	0.055	0.042
unknown	0.447	0.514	0.422	0.466
marital status: married	0.236	0.205	0.257	0.221
unmarried	0.085	0.074	0.075	0.09
unknown	0.68	0.721	0.668	0.689
dwelling type: rented	0.033	0.047	0.032	0.034
owned	0.521	0.438	0.547	0.499
unknown	0.447	0.514	0.422	0.466
income group: 1	0.041	0.05	0.055	0.035
2	0.051	0.047	0.05	0.051
3	0.05	0.037	0.046	0.051
4	0.114	0.1	0.115	0.112
5	0.139	0.114	0.149	0.131
6	0.062	0.057	0.065	0.059
7	0.023	0.018	0.022	0.023
8	0.031	0.017	0.031	0.029
9	0.024	0.027	0.022	0.025
10	0.006	0.006	0.008	0.005
11	0.003	0.005	0.003	0.003
12	0.01	0.008	0.01	0.009
unknown	0.447	0.514	0.422	0.466
coupons redeemed	0.031	0.005	0.043	0.022
