# Supplementary figures and images for: How causal machine learning can leverage marketing strategies: Assessing and improving the performance of a coupon campaign
Source: PLoS One. 2023 Jan 11;18(1):e0278937. doi: 10.1371/journal.pone.0278937 (PMC9833560; doi:10.1371/journal.pone.0278937)

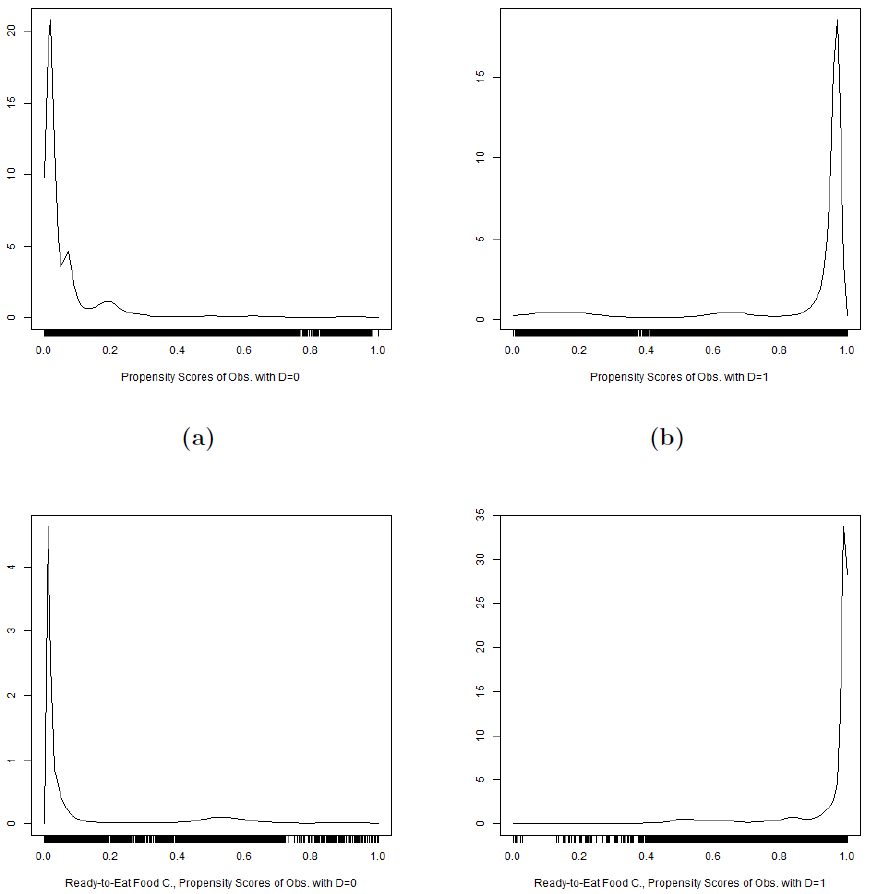

Supplement: S1 Fig — Distribution of propensity scores of receiving any coupon among observations that received (a) no coupon and (b) any coupon, as well as that of the propensity scores of receiving ready-to-eat food coupons among observations that (c) did not and (d) did receive ready-to-eat food coupons. The plots are produced with the logspline command in R with the lower and upper bounds of the support of the propensity scores are set to 0 and 1. (TIF) [file pone.0278937.s003.tif]

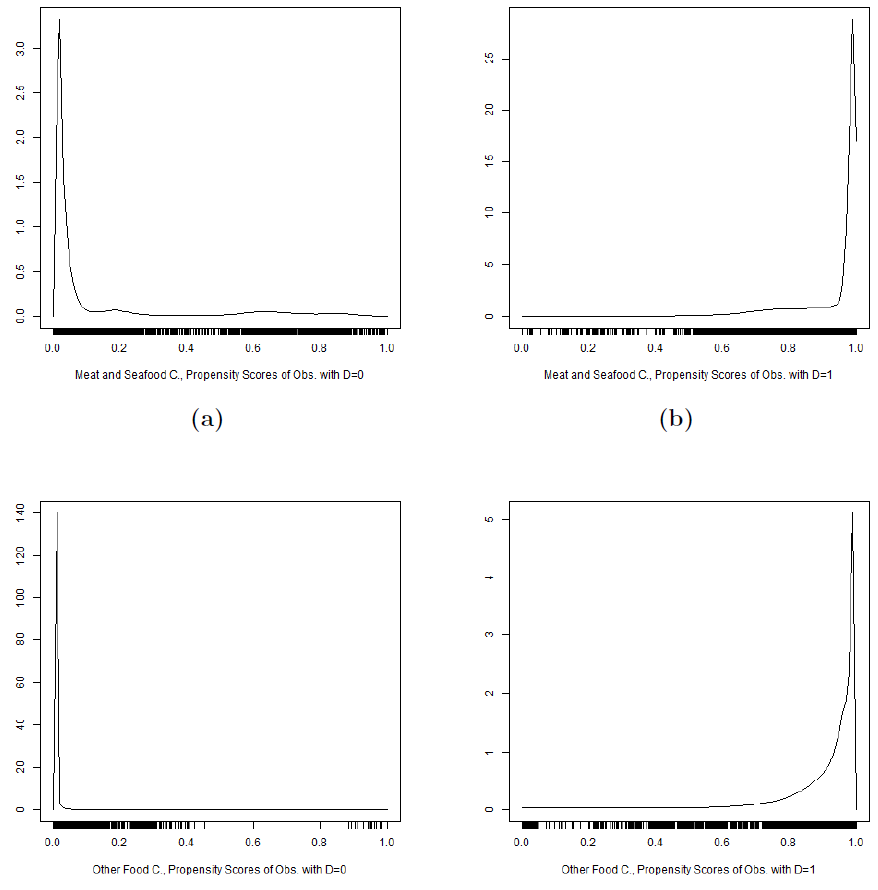

Supplement: S2 Fig — Distribution of propensity scores of receiving meat/seafood coupons among observations that (a) did not and (b) did receive meat/seafood coupons, as well as that of the propensity scores of receiving other food coupons among observations that (c) did not and (d) did receive other food coupons. The plots are produced with the logspline command in R with the lower and upper bounds of the support of the propensity scores are set to 0 and 1. (TIF) [file pone.0278937.s004.tif]

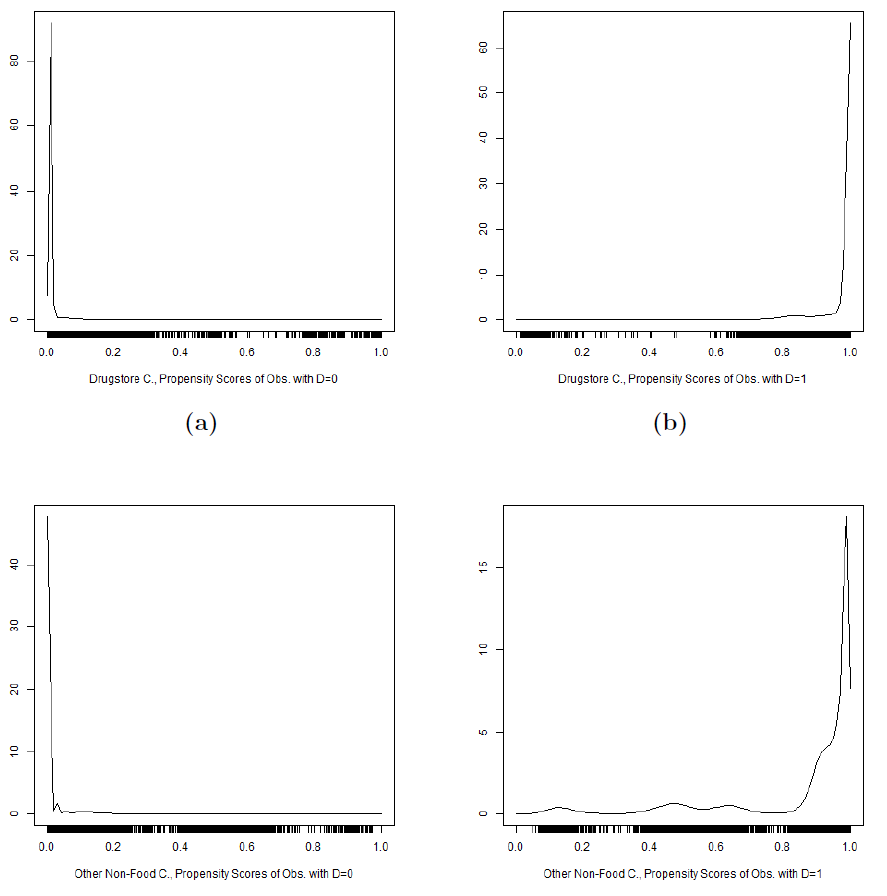

Supplement: S3 Fig — Distribution of propensity scores of receiving drugstore coupons among observations that (a) did not and (b) did receive drugstore coupons, as well as that of the propensity scores of receiving other non-food coupons among observations that (c) did not and (d) did receive other non-food coupons. The plots are produced with the logspline command in R with the lower and upper bounds of the support of the propensity scores are set to 0 and 1. (TIF) [file pone.0278937.s005.tif]

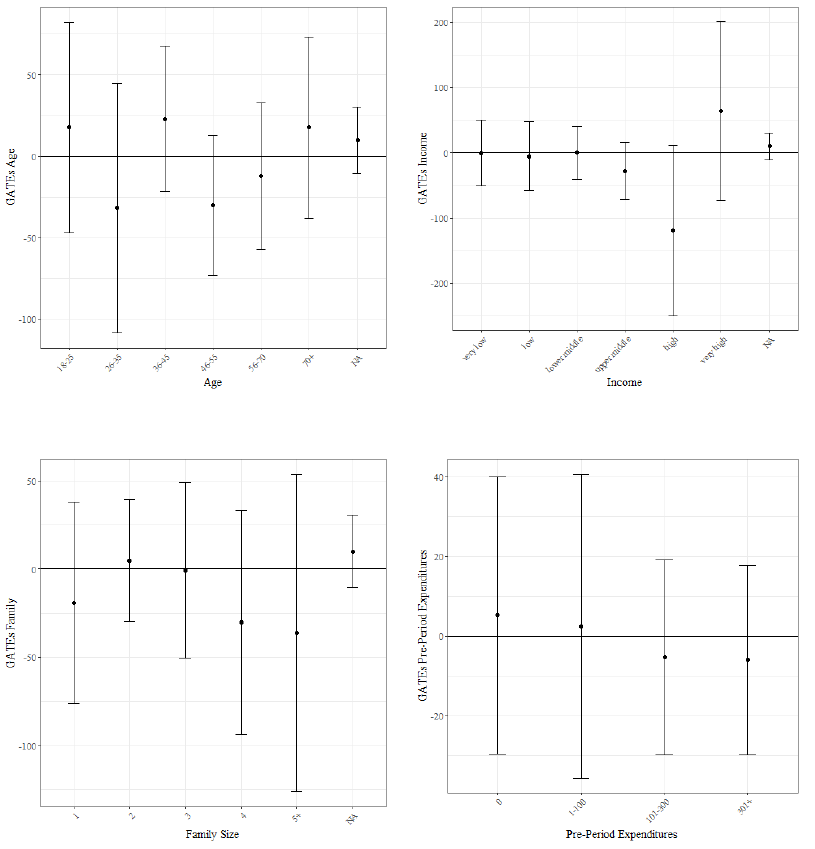

Supplement: S4 Fig — GATE estimates of ready-to-eat food coupons with 95% confidence interval, denoted in monetary units. (TIF) [file pone.0278937.s006.tif]

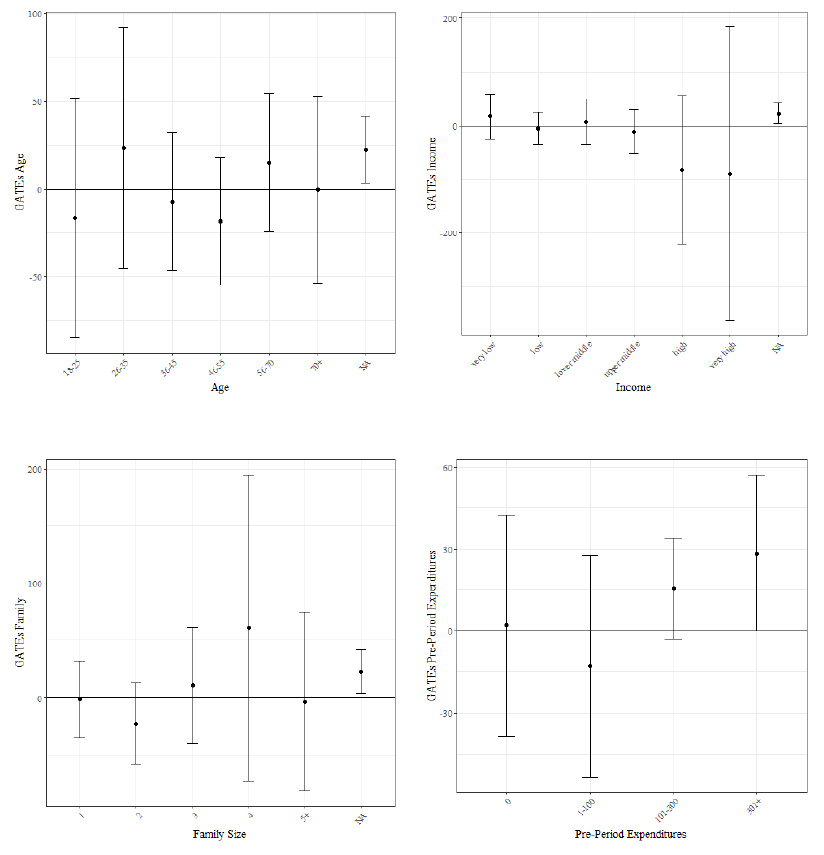

Supplement: S5 Fig — GATE estimates of meat/seafood coupons with 95% confidence interval, denoted in monetary units. (TIF) [file pone.0278937.s007.tif]

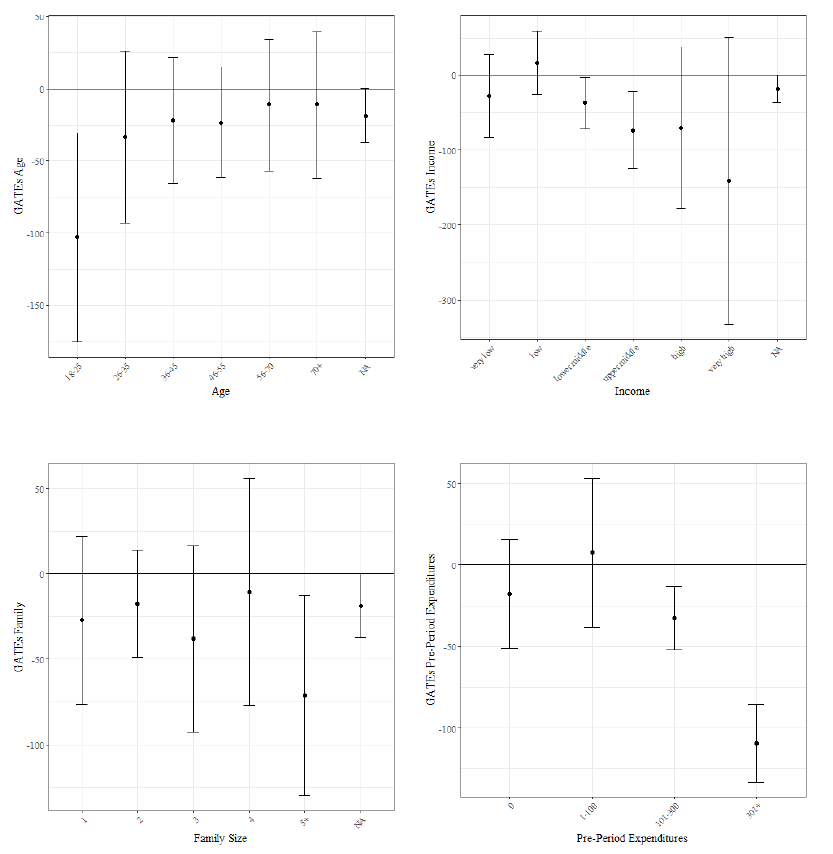

Supplement: S6 Fig — GATE estimates of coupons applicable to other non-food products with 95% confidence interval, denoted in monetary units. (TIF) [file pone.0278937.s008.tif]

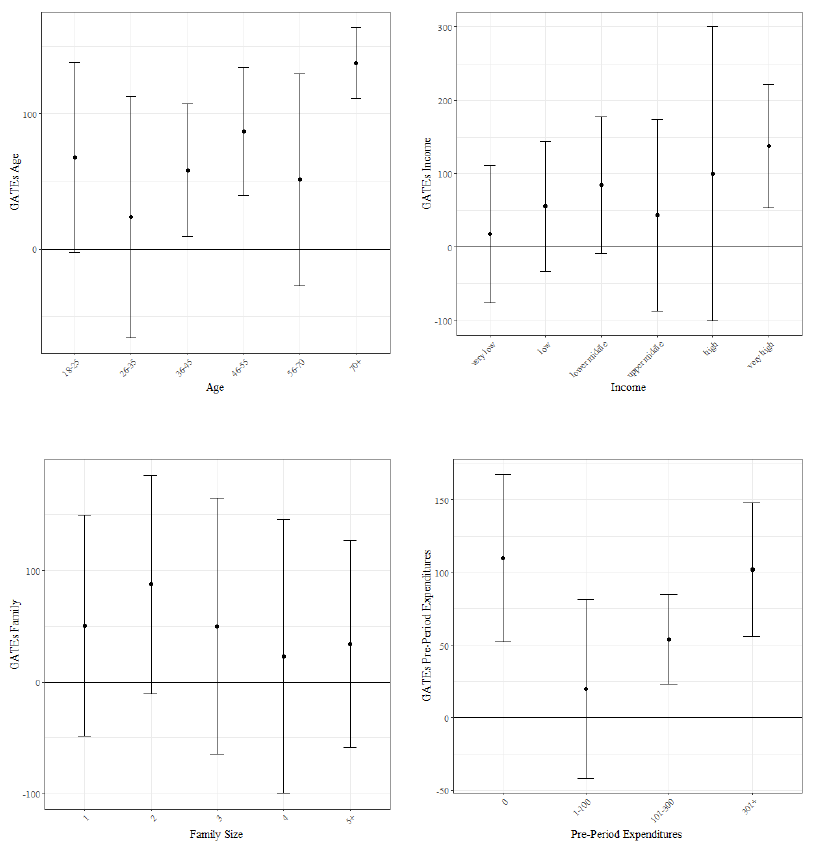

Supplement: S7 Fig — GATE estimates of receiving any coupon with 95% confidence interval, estimated in reduced data set and denoted in monetary units. (TIF) [file pone.0278937.s009.tif]

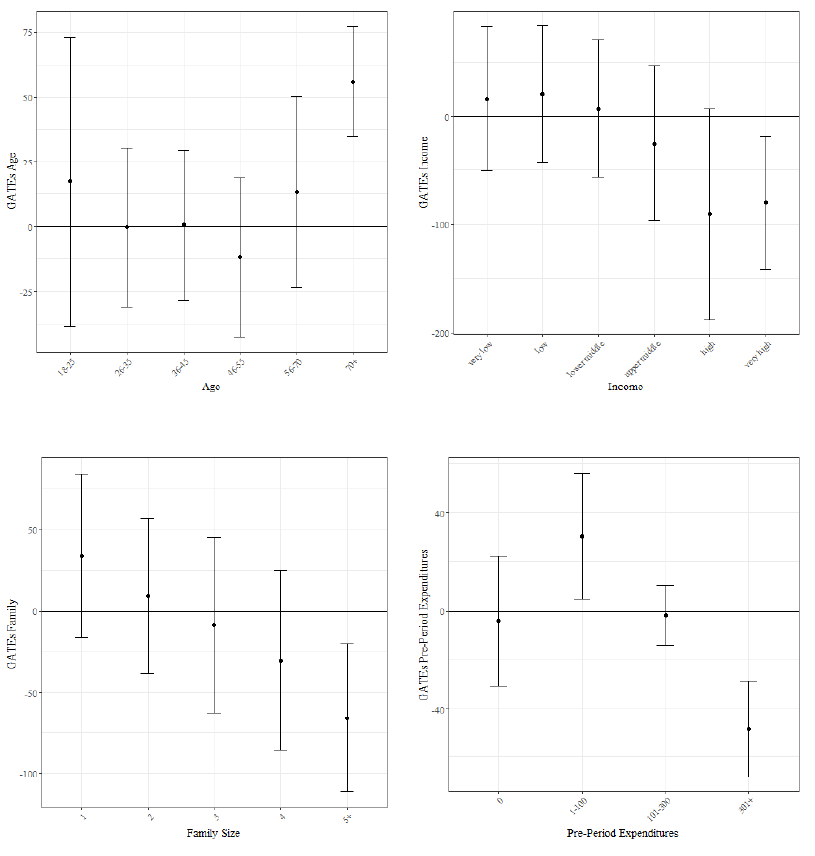

Supplement: S8 Fig — GATE estimates of ready-to-eat food coupons with 95% confidence interval, estimated in reduced data set and denoted in monetary units. (TIF) [file pone.0278937.s010.tif]

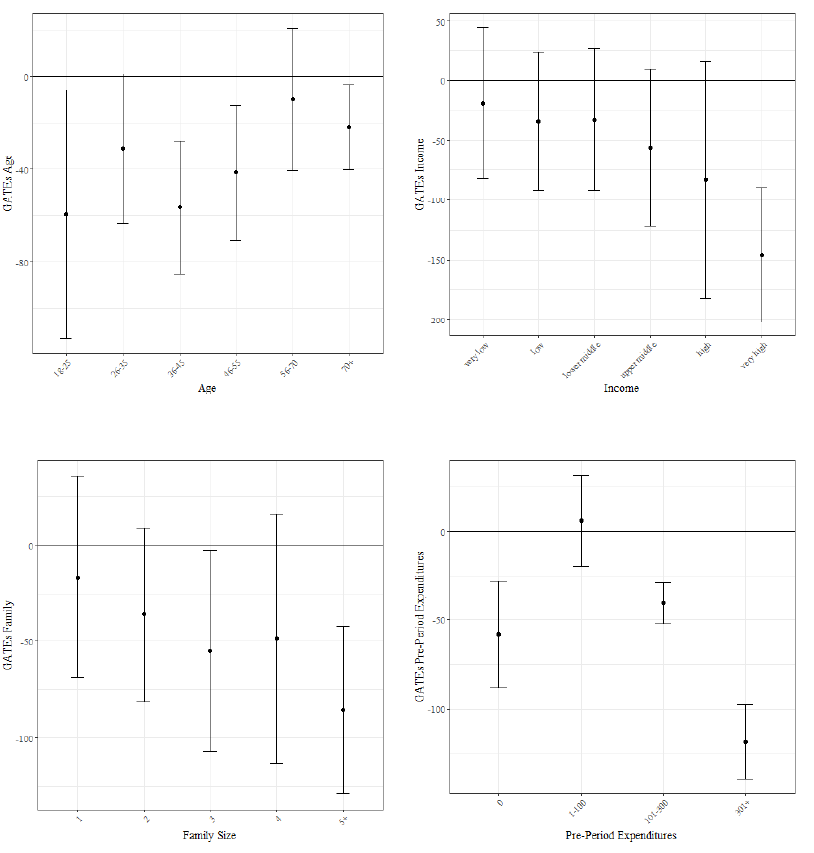

Supplement: S9 Fig — GATEs of meat and seafood coupons with 95% confidence interval, estimated in reduced data set and denoted in monetary units. (TIF) [file pone.0278937.s011.tif]

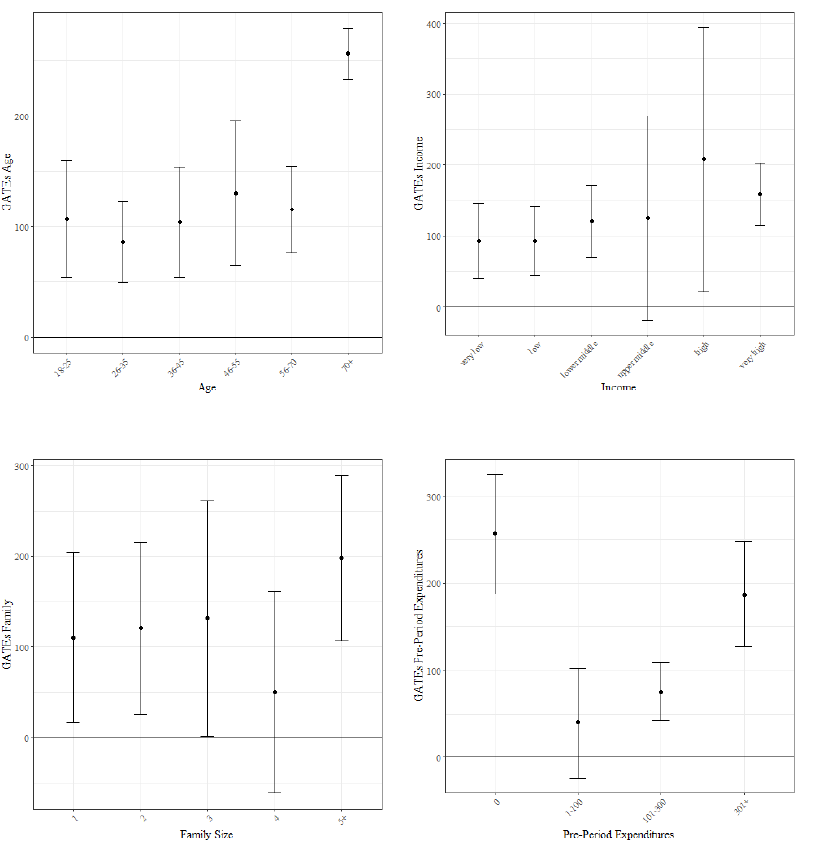

Supplement: S10 Fig — GATE estimates of coupons applicable to other food items with 95% confidence interval, estimated in reduced data set and denoted in monetary units. (TIF) [file pone.0278937.s012.tif]

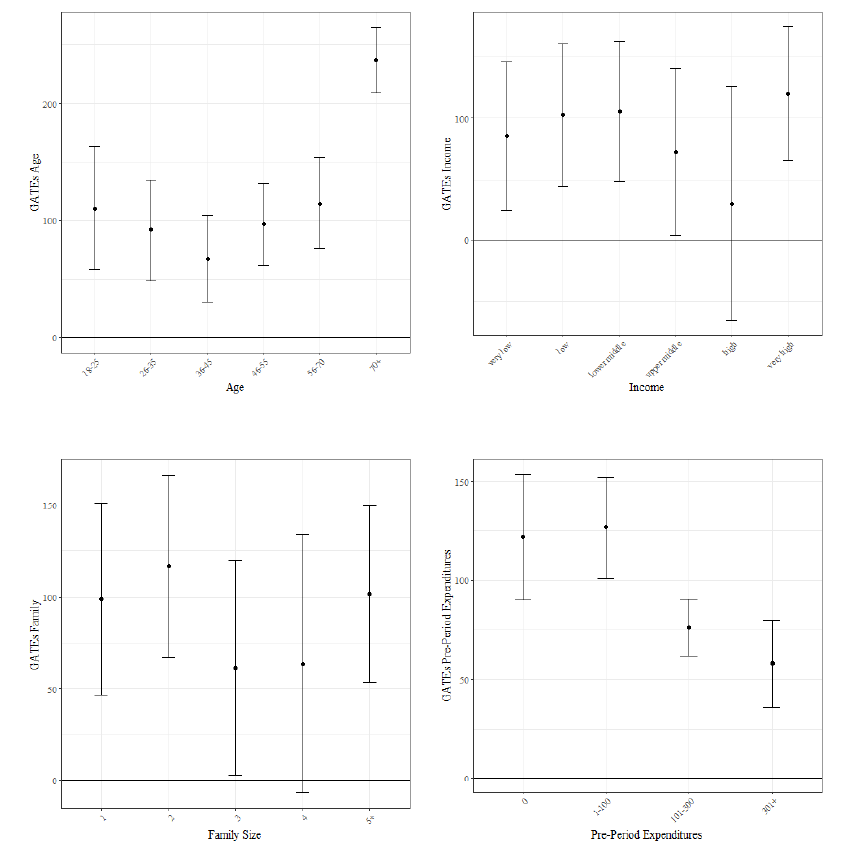

Supplement: S11 Fig — GATE estimates of drugstore coupons with 95% confidence interval, estimated in reduced data set and denoted in monetary units. (TIF) [file pone.0278937.s013.tif]

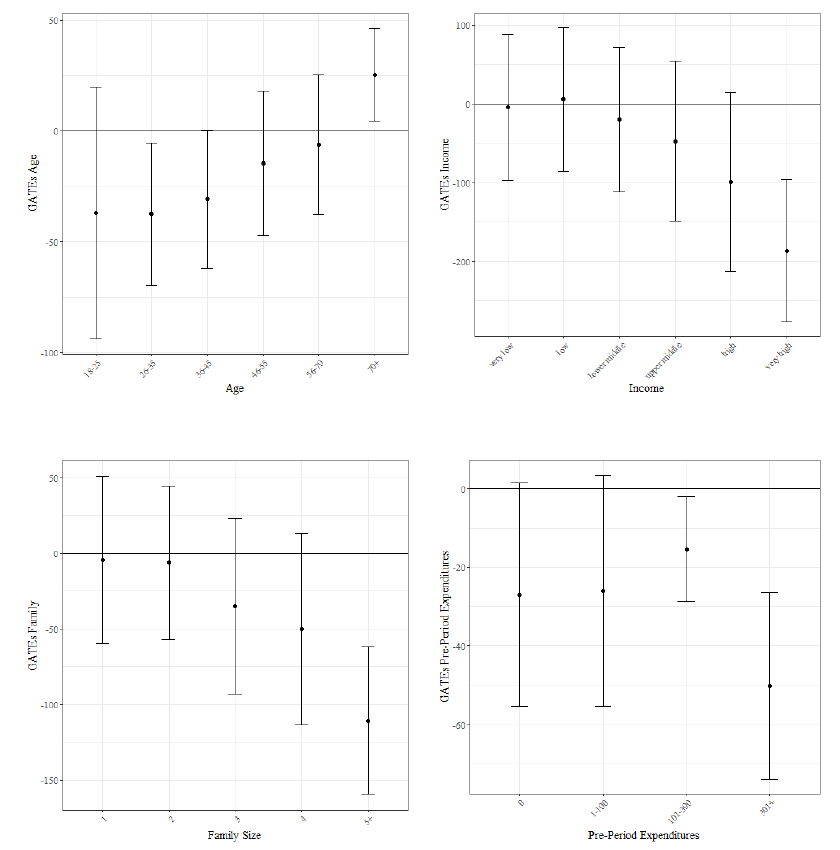

Supplement: S12 Fig — GATE estimates of coupons applicable to other non-food items with 95% confidence interval, estimated in reduced data set and denoted in monetary units. (TIF) [file pone.0278937.s014.tif]

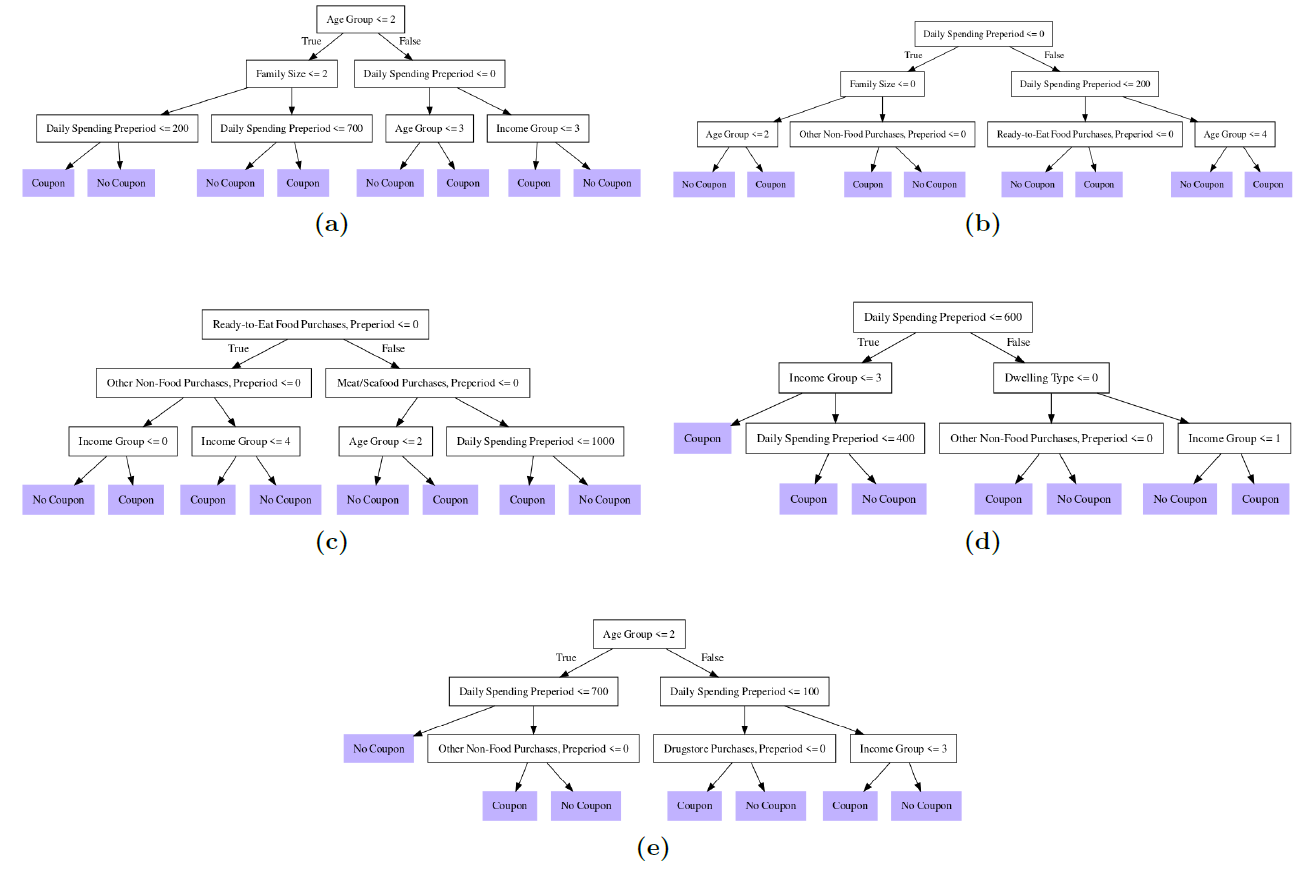

Supplement: S13 Fig — Depth-3 trees for optimally distributing coupons applicable to (a) ready-to-eat food, (b) meat and seafood, (c) other food, (d) drugstore products and (e) other non-food products, estimated in reduced data set. (TIF) [file pone.0278937.s015.tif]
